# High-Performance Carbon Nanotube Electronic Devices: Progress and Challenges

**DOI:** 10.3390/mi16050554

**Published:** 2025-05-01

**Authors:** Zirui Zhang, Nie Zhang, Zhiyong Zhang

**Affiliations:** Key Laboratory for the Physics and Chemistry of Nanodevices, Center for Carbon-Based Electronics, School of Electronics, Peking University, Beijing 100871, China; zrzhang@stu.pku.edu.cn (Z.Z.); nzhang@stu.pku.edu.cn (N.Z.)

**Keywords:** carbon nanotubes, high-performance electronics, CMOS, post silicon, integration

## Abstract

As silicon-based complementary metal-oxide-semiconductor (CMOS) technology approaches its physical and scaling limits at sub-3-nanometer nodes, critical challenges including the short-channel effect (SCE), surging power consumption, and aggravated parasitic effects have severely constrained further improvements in device performance, integration density, and energy efficiency. Carbon nanotubes (CNTs), with their superior electrical properties, exceptional gate controllability enabled by one-dimensional nanostructure, and compatibility with existing semiconductor processes, have emerged as an ideal candidate material for post-silicon high-performance electronics. Since their discovery, CNT electronics have evolved from fundamental research to a comprehensive technological framework. This review first systematically elaborates the physical characteristics of CNTs and operation mechanisms of electronic devices. Subsequently, we comprehensively summarize recent research progress in high-performance CNT electronic devices with particular emphasis on their breakthrough achievements. Through critical analysis of current developments, we thoroughly discuss fundamental challenges in material synthesis, device fabrication, and circuit integration, while evaluating potential solutions. Finally, we concentrate on future development directions for high-performance CNT devices, aiming to call for collaborative efforts from both academia and industry to accelerate the transition of CNT electronics from laboratory research to industrial implementation.

## 1. Introduction

Since the 1960s, silicon-based CMOS technology has been the cornerstone of the electronics and integrated circuit (IC) industry, driving advancements in computational power and energy efficiency in accordance with Moore’s Law. However, as technology nodes approach 3 nm and beyond, silicon-based CMOS technology faces significant challenges, including the SCE, power consumption barriers, and stronger parasitics, which hinder its ability to meet the demands of future high-performance computing (HPC), artificial intelligence (AI), and low-power embedded systems [1,2,3].

The SCE is a primary bottleneck in CMOS scaling. When the gate length (*L*_g_) is reduced below 10 nm, the channel control capability of silicon-based field-effect transistors (FETs) diminishes, leading to increased off-state leakage current (*I*_off_) and difficulty in achieving subthreshold swing (SS) below the thermodynamic limit of 60 mV/decade, thereby compromising switching characteristics. To mitigate the SCE, advanced structures such as FinFETs and gate-all-around (GAA) FETs have been developed, but these solutions increase manufacturing complexity and cost. Additionally, power consumption remains a critical issue. In HPC and data center-related applications, power consumption primarily stems from dynamic power (CV^2^f) and static leakage power. As CMOS technology operates at ultra-low voltages (*V*_DD_ < 0.7 V), the drive current (*I*_on_) of silicon-based devices decreases, while leakage current rises due to the SCE, reducing overall energy efficiency.

In summary, future high-performance electronic devices must exhibit superior SCE immunity, higher drive current, lower power consumption, and greater integration density. Traditional silicon-based CMOS technology faces severe physical limitations below 3 nm, whereas CNTs, as quasi-one-dimensional semiconductor materials, offer high mobility, symmetric conduction bands, and dangling bond-free electronic properties. CNTs also provide advantages such as low power consumption, process compatibility, and the ability to operate without doping, making them a highly competitive alternative to silicon in the post-Moore era [4,5,6,7].

Since their discovery by Sumio Iijima in 1991, CNTs have undergone several key stages in electronic applications, from fundamental exploration to device optimization [8]. In 1998, the teams from Delft University of Technology and IBM independently fabricated the first CNT field-effect transistors [9,10]. However, due to Schottky barrier issues, these devices exhibited low on-state currents, limiting their competitiveness with traditional silicon-based transistors. In 2003, researchers discovered that using high-work-function metals such as palladium (Pd) as source/drain electrodes could form barrier-free ohmic contacts for p-type CNT ballistic FETs, improving on-state conductivity [11]. This marked a critical milestone in CNT electronics. However, the realization of CNT CMOS logic circuits remained challenging due to the lack of optimized ohmic contacts for n-type CNT FETs. In 2007, the team from Peking University proposed using low-work-function metals such as scandium (Sc) as source/drain electrodes, successfully fabricating high-performance n-type CNT ballistic FETs and enabling CNT CMOS complementary logic circuits [12]. In the 2010s, advancements in high-purity semiconductor-grade CNT extraction techniques and the development of high-density, aligned CNT arrays (A-CNTs) provided the necessary support for CNT device integration. In 2020, the team from Peking University further demonstrated a 5-stage ring oscillator (RO) based on CNT FETs, achieving an operating frequency exceeding 8 GHz, showcasing the potential of CNTs in high-performance logic circuits [13]. Recently, companies such as IBM and TSMC have begun exploring CNT CMOS technology for advanced nodes, investigating its potential in HPC, low-power memory, and 3D integration [14,15]. With continuous advancements in manufacturing, CNT electronics is transitioning from laboratory research to industrialization, emerging as a key direction in future semiconductor technology.

The rapid development of carbon-based electronics and the advent of its engineering milestones have made it imperative to conduct a comprehensive review of high-performance CNT electronic devices. This paper aims to systematically outline the research progress and technological breakthroughs in CNT electronic devices. Starting from the fundamental properties of CNTs, we delve into the operational mechanisms of high-performance transistors, key technological advancements, technical challenges, and future development directions. Specifically, it covers the structural, electrical, thermal, and mechanical properties of CNTs, analyzes the physical mechanisms of high-performance transistors, reviews the latest advances in field-effect transistors, radiofrequency devices, logic circuits, 3D integration technologies, neuromorphic devices, optoelectronic integration, and novel Dirac-source transistors, while addressing critical challenges in material preparation, device structure optimization, and integration processes. Finally, it provides an outlook on future development pathways and an industrialization roadmap, offering insights for further breakthroughs in carbon-based electronics.

## 2. Fundamental Properties of CNTs

### 2.1. Structural Properties of CNTs

**Figure 1:**
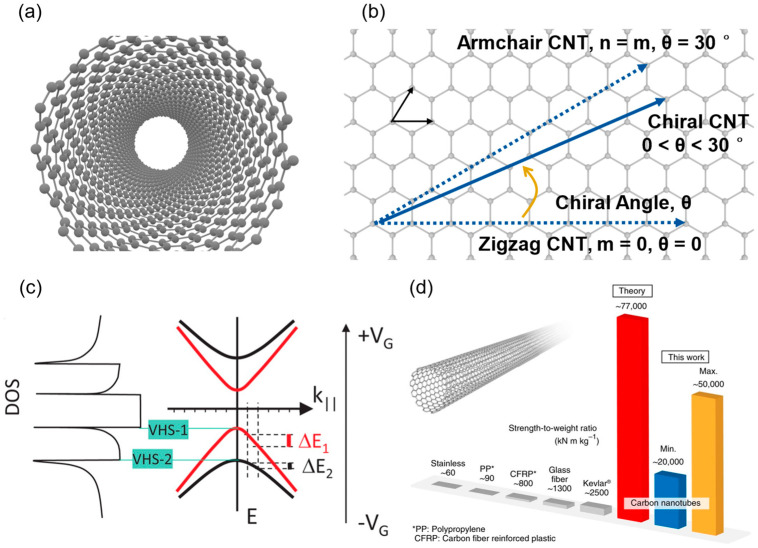
Fundamental properties of carbon nanotubes. (**a**) Schematic picture of semiconducting SWCNT. (**b**) Chirality of SWCNTs. (**c**) Density of states and band structure of semiconducting SWCNT. Reprinted with permission from [16]. Copyright 2015 American Chemical Society. (**d**) Comparison of strength-to-weight ratio of various materials. Reproduced with permission from [17]; published by Springer Nature, 2019.

Carbon nanotubes, as quasi-one-dimensional nanomaterials composed of sp^2^ hybridized carbon atoms (see Figure 1a), exhibit structural properties that underpin their potential in electronic devices. Based on the number of walls, CNTs are classified into single-walled carbon nanotubes (SWCNTs) and multi-walled carbon nanotubes (MWCNTs). SWCNTs can be viewed as rolled-up single-layer graphene, with diameters typically ranging from 0.4 to 3 nm; see Figure 1b. Additionally, CNTs exhibit semiconducting or metallic properties depending on their chirality, with approximately two-thirds being semiconducting, suitable for channel materials, and the remaining one-third being metallic, often used in interconnects. Given their higher uniformity, controllability, and ultrathin characteristics, SWCNTs are ideal materials in the post-Moore era. This review will focus primarily on high-performance CNT devices.

### 2.2. Electrical Properties of CNTs

The electrical properties of SWCNTs are central to their potential as high-performance electronic materials. First, their high carrier mobility is particularly noteworthy, with theoretical values reaching up to 10^5^ cm^2^·V^−1^·s^−1^, far exceeding those of silicon (1400 cm^2^·V^−1^·s^−1^) and III-V compounds (e.g., InGaAs~10,000 cm^2^·V^−1^·s^−1^) [18]. Experimentally, aligned SWCNTs have demonstrated field-effect mobilities as high as 3000 cm^2^·V^−1^·s^−1^, and even at gate lengths below 10 nm, mobilities remain above 200 cm^2^·V^−1^·s^−1^, comparable to silicon-based devices at 3 nm node [19,20]. This high mobility arises from their quasi-one-dimensional structure and ballistic transport characteristics, featuring a dangling bond-free surface (sp^2^ hybridization) and long mean free paths (200–500 nm at low fields, 10–15 nm at high fields), which reduce carrier scattering losses and enable quasi-ballistic transport at sub-10 nm scales, lowering dynamic power consumption [21].

Second, the ultrathin characteristics of SWCNTs enhance gate control efficiency. Notably, the natural length of CNTs (i.e., the electrostatic screening length) typically ranges from 0.2 to 1 nm [22]. This ultrashort length allows the gate electric field to efficiently shield and modulate channel carriers within a minimal spatial scale, markedly enhancing gate control efficiency. This property enables CNTs to exhibit superior performance in short-channel devices, maintaining effective gate control at nanoscale dimensions and mitigating the performance degradation caused by the SCE in traditional silicon devices. The current density of the 200 nm gate length of the carbon nanotube transistor can reach 1.18 mA/μm and the gate delay of the ring oscillator circuit can be as low as 11.3 ps at an operating voltage of 1 V, which exceeds the performance of silicon-based devices of the same size [23]. In addition, the low-dimensional characteristics bring about significant quantum effects, such as low quantum capacitance, compared to the capacitance of bulk materials, which further reduces the operating voltage of the device (to as low as 0.4 V), achieving a balance between high performance and low power consumption [12]. Experimentally, 5 nm gate-length CNT FETs have achieved *SS* values of 73 mV/dec, approaching the ideal value (60 mV/dec), while silicon-based GAA devices at the same scale exhibit *SS* of 85 mV/dec. These properties position SWCNTs as an ideal candidate to surpass silicon-based CMOS [7,24]. Additionally, the ambipolar nature of CNTs can be effectively tuned by gate voltage under specific conditions, offering greater flexibility in device design. Together, these attributes endow CNTs with exceptional gate control in theory, laying a crucial foundation for the development of high-performance, low-power electronic devices.

The 1D quantum confinement effect in CNTs results in a van Hove singularity distribution of density of states as shown in Figure 1c, with only a few subbands excited at low bias, avoiding multi-subband scattering in traditional semiconductors and enabling quantized conductance (G_0_ = 2*e*^2^/*h* ≈ 12.9 kΩ^−1^) [12,16]. Experimentally, single s-SWCNT FETs have achieved on-state conductance of 0.7G_0_, approaching the theoretical limit [25]. Additionally, CNTs exhibit no Fermi-level pinning at metal contacts, and by selecting metals with appropriate work functions (e.g., Pd for p-type, Sc for n-type), ideal ohmic contacts can be formed, further enhancing carrier injection efficiency. This unique property enables low contact resistance (~36 kΩ/tube) even at 10 nm contact lengths [26].

### 2.3. Thermal and Mechanical Properties of CNT

In addition to electrical properties, the thermal and mechanical properties of SWCNTs also support their application in high-performance electronic devices. Their thermal conductivity is exceptionally high, with axial values reaching up to 6000 W/(m·K), much higher than copper’s 385 W/(m·K), aiding in heat dissipation during high-temperature operation [27,28,29]. According to Figure 1d, SWCNTs exhibit ultrahigh strength and flexibility, with Young’s moduli of up to 100 GPa and fracture strains of 20%, making them suitable for flexible electronics [17,30]. In terms of thermal budget, CNT-based devices support low-temperature fabrication (<400 °C), enabling monolithic 3D integration (M3D) [31].

## 3. Physical Mechanisms of High-Performance CNT Devices

### 3.1. Types of Channel Materials

**Figure 2:**
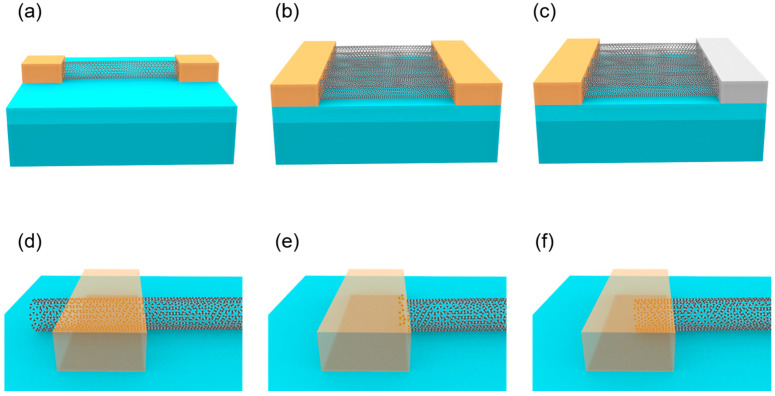
Device structures and contact types of CNT devices. (**a**) Single CNT-based FET. (**b**) A-CNT FET. (**c**) A-CNT diode with asymmetric contacts. (**d**) Side contact. (**e**) End contact. Black dots near the semiconductor–metal interface correspond to the formation of carbide. (**f**) Full contact.

In carbon nanotube-based electronic devices, the type of channel material is critical to device performance. CNT channel materials are mainly classified into three forms: single carbon nanotube, CNT networks, and CNT arrays; see Figure 2a,b. Single CNTs offer extremely high intrinsic mobility but are primarily used in early theoretical studies and prototype demonstrations. CNT networks, formed by randomly oriented CNTs, suffer from performance inconsistency due to inter-tube contact resistance and random distribution, making them inadequate for high-performance devices. In contrast, CNT arrays, composed of high-density, highly aligned carbon nanotubes, retain the high mobility of single CNTs while reducing inter-tube contact resistance through alignment, improving device uniformity and reproducibility. Therefore, for high-performance CNT electronic devices, particularly those targeting low power, high speed, and high integration, high-density and highly aligned CNT arrays are the optimal choices.

### 3.2. Types of Contact

According to the working mechanisms of electronic devices, there exist two main structures for high-performance CNT electronics, which are field effect transistors (see Figure 2a,b) with symmetric contacts and diodes (see Figure 2c) with asymmetric contacts. The performance of CNT electronic devices largely depends on the contact properties at the metal–semiconductor interface, where forming ohmic contact is crucial to leveraging their high mobility advantage. Specifically, silicon-based metal-oxide-semiconductor FETs (MOSFETs) and diodes rely on doping optimization to form ohmic contacts, avoiding tunneling limitations, but doping uniformity becomes difficult to control below 5 nm nodes, highlighting the unique advantages of CNT devices in contact engineering.

The type of contact in CNT devices affects their electrical performance, primarily categorized as side contact (see Figure 2d) and end contact (see Figure 2e). Side contact involves metal overlaying the CNT sidewall, relying on van der Waals (vdW) forces or weak chemisorption. It features a large contact area, though with a high barrier, of which the resistance reaches 90 kΩ per tube at a contact length of approximately 20 nm [32]. In contrast to side contact, end contact entails metal bonding directly to the sp^2^ carbon atoms at CNT ends, forming covalent connections that substantially reduce the Schottky barrier and contact resistance. Through end-bonded contact techniques, the contact resistance of A-CNT devices can be effectively reduced. For instance, the contact resistance of a single tube with molybdenum carbide end contact remains around 36 kΩ even at a contact length of 10 nm [26]. The superiority of end contact enhances the potential of CNT devices for high-performance applications, though challenges in process complexity and stability remain to be addressed. Combining the merits of side-contact and end-contact configurations, researchers have introduced the concept of full contact, as shown in Figure 2f. Unlike side contact, which interfaces solely with the nanotube sidewall, or end-contact, which connects only to the nanotube end bonds, full contact relies on an etching process to define the channel length. This enables the contact metal to simultaneously envelop both the sidewall and tube end of the carbon nanotube. Consequently, this approach reduces contact resistance, enhances on-state current and transconductance, and mitigates the dependence of contact resistance on contact length, thereby facilitating device scaling. However, the fabrication precision required for full contact exceeds that of side contact, but it eliminates the need for the high-temperature annealing inherent to end-contact processes [33].

As integrated circuit technology advances and transistor sizes continue to shrink, the impact of contact resistance on device performance becomes increasingly significant. During scaling, channel resistance decreases with reduced channel length, but contact resistance rises due to shorter electrode lengths. For CNT electronic devices, contact resistance comprises two components: the fundamental contact resistance determined by quantum conductance and the non-penetrating contact resistance introduced by fabrication processes. Ideally, the interface between a ballistic mesoscopic one-dimensional conductor and a macroscopic metal electrode introduces a fundamental contact resistance governed by the number of channel modes (*N*_ch_), with a theoretical value of 12.9 kΩ/*N*_ch_. For SWCNTs, where *N*_ch_ equals 2, this yields a fundamental contact resistance of approximately 6.45 kΩ. However, in practical devices, imperfect fabrication often results in non-penetrating contacts, adding extra resistance tied to carrier transmission and contact length. For instance, experimental side-contact configurations typically exhibit total contact resistance of 10–15 kΩ [12,34,35,36]. To meet the demands of advanced nodes, multiple CNTs must be placed in parallel within the channel to reduce contact resistance per unit length. The International Roadmap for Devices and Systems (IRDS) indicates that, beyond 2031, the critical contact dimension for digital logic transistors should shrink to 18 nm, with contact resistance below 232 Ω [3]. Theoretical analysis suggests that an array of CNTs with a density of 40–70 tubes/μm is required as the channel material, though inter-tube interactions complicate the practical scenario.

Specifically, compared to silicon-based MOSFETs, CNT FETs exhibit significant differences and potential in their physical mechanisms. Silicon MOSFETs rely on carrier transport in bulk materials, with mobility and subthreshold swing limited by three-dimensional scattering and the Boltzmann constraint, resulting in notable performance degradation below the 5 nm node. However, silicon MOSFETs retain advantages in process maturity and leakage current control, as their larger bandgap (1.1 eV) effectively suppresses gate-induced drain leakage (GIDL), whereas the 0.4–0.7 eV bandgap of CNT FETs poses challenges for off-state power consumption. Overall, CNT FETs outperform silicon-based technology in high performance, low power, and scaling capability, yet optimizing contact resistance and suppressing leakage current remain critical hurdles for their practical application.

## 4. Key Advances in High-Performance CNT Electronics

### 4.1. CNT Field-Effect Transistors

As the core devices of carbon-based electronics, CNT FETs have made significant progress in design and fabrication in recent years, particularly demonstrating the potential to surpass silicon-based technology in high-performance applications. The important metrics for CNT FETs are listed in Table 1 for reference. In the following part, we will discuss the development of CNT FETs based on these metrics.

**Figure 3:**
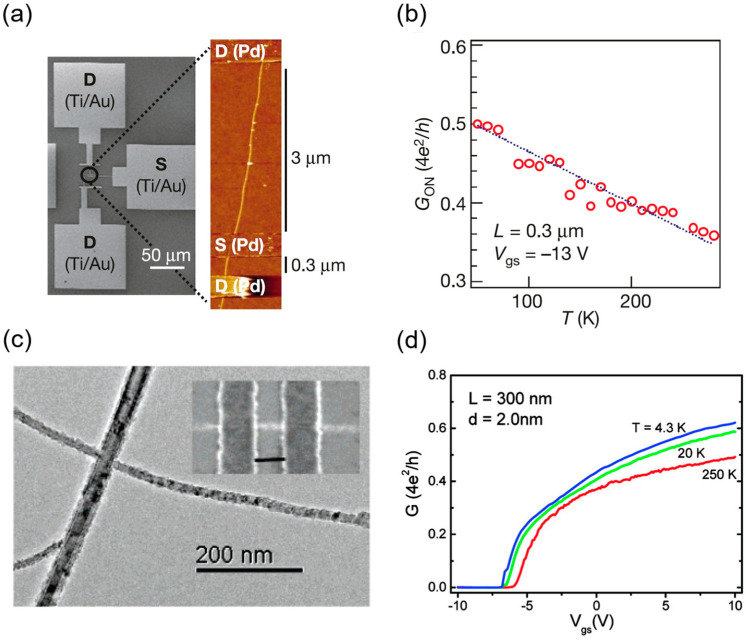
Ballistic FETs built on single CNTs. (**a**,**b**) P-type ballistic CNT FET and its conductance [11], reproduced with the permission from SNCSC. (**c**,**d**) N-type ballistic CNT FET and its conductance. Reprinted with permission from [12]. Copyright 2007 American Chemical Society.

**Figure 4:**
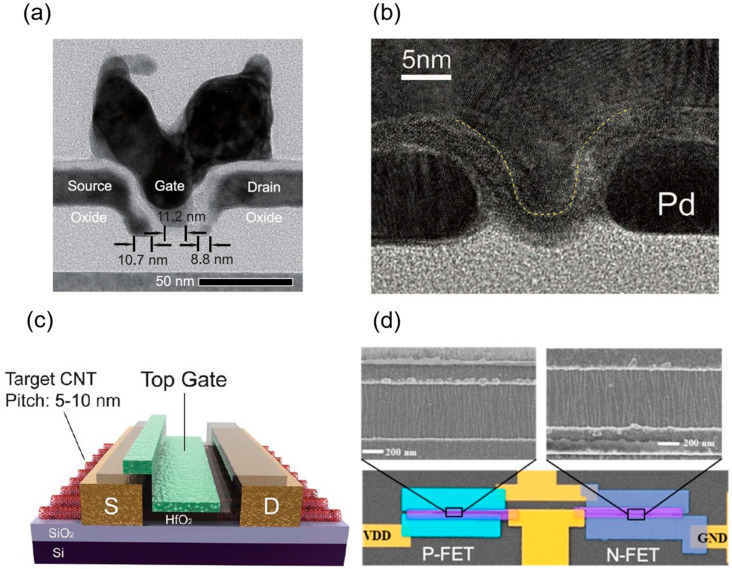
High-performance CNT FETs. (**a**) P-channel transistor footprint scaled to 40 nm. From [15]. Reprinted with permission from AAAS. (**b**) SEM micrograph of the CNT FET with gate length of 5 nm. From [7]. Reprinted with permission from AAAS. (**c**) First demonstration of A-CNT FETs built on ideal CNT arrays with tunable density of 100~200 tubes per micrometer and semiconducting purity estimated to be over 99.9999%. From [13]. Reprinted with permission from AAAS. (**d**) Complementary transistors based on A-CNTs with the best n-type CNT FETs to date. Reprinted with permission from [39]. Copyright 2022 American Chemical Society.

Javey et al. at Stanford University first achieved a high-performance P-type ballistic CNT transistor with an on-state conductance as high as 0.65G_0_ in 2003 (view Figure 3a,b) [11]. In 2007, the Peking University team utilized scandium (Sc) as a contact metal to achieve the first barrier-free N-type ohmic contact with an on-state conductance of 0.5G_0_ at room temperature (see Figure 3c,d), and in 2008, they demonstrated a high-performance N-type device at 120 nm gate length with gate delay as low as 0.86 ps, marking the first showcase of CNT FETs’ terahertz speed potential [12,37]. In 2010, Franklin and Chen from IBM further scaled the channel length of a P-type CNT FET to 15 nm, achieving an on-state conductance of up to 0.7G_0_ and a peak transconductance of 40 μS, setting a new performance benchmark for single-tube devices [38]. Subsequently, they achieved a subthreshold swing of 94 mV/dec in a P-type CNT FET with a sub-10 nm gate length, highlighting exceptional resistance to short-channel effects and superior scaling potential [25]. In 2017, Cao et al. employed a low-temperature process (650 °C) with end contact technology and atomic layer deposition (ALD)-grown aluminum oxide gate dielectric to develop a P-type CNT device with contact and channel lengths of approximately 10 nm, shown in Figure 4a, matching the overall size of the silicon-based 5 nm technology node. At an operating voltage of 0.5 V, this device achieved a normalized on-state current of 700–900 μA/μm, a subthreshold swing of about 85 mV/dec, and an off-state leakage current of only 4 nA, delivering a performance advantage twice that of advanced silicon nodes while consuming roughly half the energy [15]. In the same year, the group from Peking University reported P-type and N-type CNT FETs at a 10 nm gate length and 0.4 V operating voltage, with on-state currents of 17.5 μA and 20 μA, respectively, outperforming silicon-based devices of the same size in normalized metrics. At a 5 nm gate length (see Figure 4b), a P-type CNT FET with graphene as the contact electrode maintained robust gate control, achieving an intrinsic gate delay of 43 fs, over twice as low as that of silicon-based 10 nm nodes and approaching the theoretical limit for binary switches (40 fs), with an energy-delay product roughly an order of magnitude lower than that of comparable silicon devices [7].

Early research primarily focused on the single carbon nanotube, emphasizing the exploration of its device operating principles and ultimate performance characterization. As technology advanced and application demands evolved, the research focus gradually shifted toward network-like CNTs and array structures. Given the stringent requirements for large-area uniformity and high performance in practical applications, achieving high-performance devices relies on the controllable preparation of high-quality CNT arrays, making arrayed CNTs a central direction in current research. In 2020, Liu et al. from Peking University utilized dimension-limited self-alignment (DLSA) to prepare aligned CNTs. Their top-gate FETs (view Figure 4c) exhibited performance surpassing that of commercial silicon-based MOSFETs, achieving an *I*_on_ of 1.3 mA·μm^−1^ and a *g*_m_ of 0.9 mS·μm^−1^ [13]. In 2021, Yanxia Lin et al. from Peking University successfully fabricated enhancement-mode field-effect transistors (E-mode FETs) based on A-CNTs films for the first time, overcoming the limitations of previous A-CNT FETs, which were predominantly depletion-mode (D-mode) with poor *SS* [23]. In top-gate FETs with a 500 nm gate length, they achieved a carrier mobility of 1850 cm^2^·V^−1^·s^−1^, which approaches that of single CNTs grown by chemical vapor deposition (CVD), setting a record for A-CNT film materials. P-type A-CNT FETs with a 200 nm gate length exhibited excellent electrical performance, including an *SS* of 73·mV dec^−1^, *g*_m_ of 1 mS·μm^−1^, and *I*_on_ of 1.18 mA·μm^−1^ (at −1 V bias), surpassing commercial silicon-based transistors of the same gate length. In 2022, Chenchen Liu from Peking University addressed the performance bottleneck of aligned CNT n-type FETs, boosting *I*_on_ to 800 μA·μm^−1^ and peak *g*_m_ to 250 μS·μm^−1^, while achieving symmetric high-performance A-CNT CMOS technology for the first time, illustrated in Figure 4d [39]. In 2023, Yanxia Lin from Peking University introduced a full-contact process and scaled the contact-to-gate pitch (CGP) of A-CNT FETs to 55 nm, yielding an *I*_on_ of 3.31 mA·μm^−1^ and *g*_m_ of 2.69 mS·μm^−1^, performance comparable to 10 nm silicon-based PMOS, demonstrating CNTs’ potential at sub-10 nm nodes [33]. In 2024, Shengman Li from Stanford University further optimized NMOS FET devices. Departing from the conventional approach of using low-work-function metals like Sc for NMOS contacts, they employed an AlN_x_ doping process on dense A-CNTs to achieve high-performance NMOS, with both NMOS and PMOS delivering drive currents exceeding 300 μA·μm^−1^ at *V*_ds_ of 1 V [41]. Nathaniel Safron et al. from TSMC pioneered the application of a nanosheet structure to CNT PMOS FETs, addressing the electrostatic control challenges in high-density CNT arrays. At *V*_ds_ of −0.5 V and channel length (*L*_ch_) of 70 nm, they achieved a drive current of 1.15 mA/μm, setting a new record for CNT FET drive current performance and surpassing silicon-based FinFET structures, with an *SS* of 135 meV [40]. Meanwhile, Yifan Liu et al. from Peking University, through systematic gate interface engineering, fabricated high-performance FETs based on high-density aligned CNT (A-CNT) arrays, achieving a record-breaking peak transconductance *g*_m_ of 3.7 mS/μm for ultrathin-channel transistors, surpassing the highest transconductance of silicon planar FETs [43]. This breakthrough stemmed from improved gate stack quality, notably a reduction in interface trap density to 5.7 × 10^11^ cm^−2^·eV^−1^ via an H_2_O pre-soak process. In devices with a 100 nm gate length, the saturation *I*_on_ reached 2.45 mA/μm, with a *g*_m_/*I*_on_ ratio exceeding 1.5, reflecting excellent gate efficiency and energy efficiency. Beyond fabricating devices on silicon substrates, Xiaohan Cheng et al. from the team utilized a low-loss glass substrate, reducing parasitic capacitance and energy loss [42]. The A-CNT FETs achieved *I*_max_ exceeding 1 mA/μm and a peak transconductance of 1.3 mS/μm (*V*_ds_ = −1.0 V). Devices with a 250 nm gate length exhibited transfer characteristics nearly identical to those of 90 nm node silicon-based devices at supply voltage (*V*_DD_) of 1 V. Their ring oscillator achieved a gate delay of 9.86 ps, which is the lowest reported value among all nanomaterials at the time, even surpassing silicon-based devices at lower *V*_DD_ [42].

In summary, systematic gate interface engineering and process optimization have driven significant advances in A-CNT FET performance and scalability, paving a new technological pathway for next-generation high-performance electronic devices.

### 4.2. CNT Radiofrequency Devices

Carbon nanotube radiofrequency (RF) devices, with their exceptional high-frequency performance and low-noise characteristics, exhibit tremendous potential in wireless communication and RF circuit applications. In 2004, Peter J. Burke proposed a small-signal equivalent circuit model based on the geometric structure and physical mechanisms of CNT transistors, combined with the universal behavior of field-effect devices. This model predicted the THz-range high-frequency performance of CNT transistors, suggesting that a cutoff frequency exceeding 400 GHz could be achieved when the gate length is scaled down to 100 nm [45]. Compared to traditional bulk materials and two-dimensional materials, a key advantage of CNTs in RF applications lies in broader substrate compatibility. Conventional bulk materials typically require the growth of high-quality single-crystal substrates with doping, whereas DLSA enables direct deposition onto any insulating substrate. In contrast, epitaxial growth of traditional silicon-based materials or high-RF-performance III-V compounds directly on insulating substrates is extremely challenging, often resulting in poor material quality with numerous defects, failing to leverage the benefits of insulating substrates. Other low-dimensional materials, such as graphene, require transfer processes to be applied onto insulating substrates, but the damage incurred during transfer hampers subsequent processing and application. Thus, the unique fabrication methods of CNTs provide a distinct edge in RF applications. In the following sections, we will explore the evolution of structures in CNT RF devices and highlight their key advancements in circuit applications.

#### 4.2.1. Planar Gate Structures

The development of carbon nanotube RF devices began in 2006, with early research focusing on easily fabricated planar gate structures. Performance improvements were pursued through material purity optimization and device structure refinement. In planar gate designs, the gate electrode is positioned either above or below the channel, typically in a symmetric configuration, though asymmetric gate or source-gate structures were also explored for performance tuning. This structure, commonly used in traditional silicon-based MOSFETs and early CNT RF FETs, offers simplicity and mature fabrication processes. However, its high-frequency parasitic effects are pronounced, limiting improvements in current gain cutoff frequency and power gain cutoff frequency. In 2006, Bethoux et al. first reported an RF device based on an electrophoretically prepared CNT thin film (10 tubes/μm) with an Al_2_O_3_ back-gate structure, achieving intrinsic current/power-gain cutoff frequency (*f*_T_/*f*_MAX_) of 8 GHz/10 GHz [46]. Subsequently, Le Louarn et al. enhanced CNT alignment and density, boosting intrinsic *f*_T_ to 30 GHz and highlighting CNTs’ potential in high-frequency applications [47]. In 2009, Nougaret et al. increased semiconducting purity to 99% and refined the electrophoresis process, pushing intrinsic *f*_T_ beyond 80 GHz, approaching silicon-based RF device levels for the first time [48]. In 2012, Mathias Steiner et al. utilized a 100 nm top-gate transistor structure and electrophoretic solution self-assembly to achieve 99.6% semiconducting purity, yielding an intrinsic cutoff frequency of 153 GHz/30 GHz. This marked the first instance of CNT RF transistor *f*_T_ exceeding 100 GHz, signifying exceptional high-frequency capabilities [49]. Beyond electrophoresis, Rogers et al. have grown CNT arrays via CVD to fabricate top-gate transistors, increasing non-de-embedding *f*_T_ from 0.5 GHz to 2.5 GHz by 2008, and achieving the first CNT-based radio [50,51]. By 2009, they reached non-de-embedding *f*_T_/*f*_MAX_ of 5 GHz/9 GHz. However, enhancing RF gain necessitates higher density and purity [52].

Further advancements came through optimizing air-gap planar gate structures and material improvement. In 2019, Donglai Zhong from Peking University employed a network CNT film (50 tubes/μm) with a 20 nm air-gap top-gate structure (air-spacer top-gate structure). The resulting CNT RF transistor achieved an on-state current of 0.34 mA/μm and transconductance of 0.4 mS/μm, with an intrinsic *f*_T_ of 281 GHz at a 30 nm gate length and a de-embedded *f*_MAX_ of 190 GHz at a 90 nm gate length. This marked the first time both *f*_T_ and *f*_MAX_ of a CNT RF transistor exceeded 100 GHz, highlighting the significant potential for power gain applications [53]. As RF transistor processes improved, the high carrier mobility and low parasitic capacitance of CNTs fully showcased their high-frequency advantages, revealing substantial potential in applications like RF amplifiers and mixers. In 2021, Zhou et al. further optimized device structures and reduced gate parasitics, elevating the measured non-de-embedded *f*_MAX_ to 90 GHz. This enabled a K-band RF amplifier with high gain and linearity in the 18–27 GHz range, matching III-V RF transistors in overall performance for the first time and providing initial evidence of the commercial value of carbon-based RF electronics [54].

**Figure 5:**
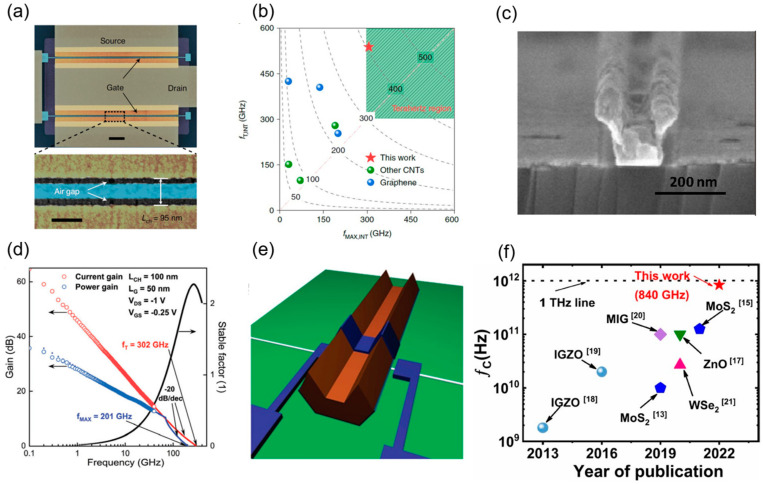
CNT RF devices. (**a**) SEM image, (**b**) Comparison of intrinsic *f*_T_/*f*_MAX_ of published CNT and graphene transistors reported by Huiwen Shi. Reproduced with permission from [55]; published by Springer Nature, 2021. (**c**) SEM characterization of T gate. Reprinted with permission from [56]. Copyright 2012 American Chemical Society. (**d**) Extraction of *f*_T_ and *f*_MAX_ with improved gate stack and interface state density as low as 5.7 × 10^11^ cm^−2^·eV^−1^ [43]. Reprinted with permission from IEEE Proceedings. (**e**) Schematic illustration of U gate. Reprinted with permission from [57]. Copyright 2011 American Chemical Society. (**f**) Benchmarking the *f*_C_ of the fabricated A-CNT SBDs with the state-of-the-art Schottky diodes comprising different materials [58]. Reprinted with permission from IEEE Electron Device Letters.

Huiwen Shi et al. from Peking University designed and optimized the A-CNT preparation process and fabricated air-gap RF devices (see Figure 5a), featuring a high maximum mobility of 1580 cm^2^·V^−1^·s^−1^ and a saturation velocity of up to 3.0 × 10^7^ cm·s^−1^. Based on that, high-performance RF transistors fabricated on a 101.6 mm quartz insulating substrate achieved extrinsic *f*_T_/*f*_MAX_ of 186 GHz/158 GHz at a 50 nm gate length. To further explore the frequency potential of CNTs, the team fabricated RF transistors on a high-resistivity silicon substrate, achieving the intrinsic *f*_T_ and *f*_MAX_ of 540 GHz and 306 GHz (see Figure 5b), respectively, at a 50 nm gate length, marking the first entry of carbon-based RF devices into the terahertz range. Their K-band amplifier demonstrated a power gain of 23.2 dB at 12 GHz, a 1 dB compression point output power greater than 9 dBm, and a third-order intercept (OIP3/P_dc_) of 19.7 dB at 31.2 dBm. These key metrics show advantages over some commercial products (e.g., HMC6981) while drastically reducing costs, underscoring the potential of carbon-based RF electronics for sixth-generation communication technologies [55].

In 2023, Jiale Qian et al. fabricated RF transistors on high-resistivity silicon using CNT arrays, achieving an extrinsic *f*_T_ of 158 GHz and *f*_MAX_ of 131 GHz. This marked the first simultaneous realization of amplification and frequency conversion in the millimeter-wave band (above 30 GHz), with a power amplification gain of 6.8 dB, a 3-dB bandwidth exceeding 40 GHz, and a gain-bandwidth product (GBW) approaching 100 GHz, outperforming other nanomaterials like graphene [59]. Meanwhile, Zhou et al. utilized UV-ozone (UVO) post-processed CNT arrays and further reduced the gate length of CNT RF FETs to 35 nm, achieving extrinsic *f*_T_ of 376 GHz and *f*_MAX_ of 318 GHz, both exceeding 300 GHz for the first time. Their K-band amplifier delivered a power gain exceeding 24 dB at 18 GHz [60]. To further reduce parasitic resistance and enhance RF performance, researchers introduced T-shaped gate structures.

#### 4.2.2. T-Shaped Gate Structures

The T-shaped gate consists of a wide top (the ‘brim’) and a narrow bottom (the part contacting the channel), resembling the letter ‘T’, illustrated in Figure 5c. The wide top reduces gate resistance, while the narrow bottom maintains gate control over the channel and minimizes gate-to-source (*C*_gs_) and gate-to-drain (*C*_gd_) parasitic capacitances, further enhancing the overall performance of RF devices. Several research groups have explored the performance improvements offered by this structure. In 2012, Yuchi Che from the University of Southern California developed a self-aligned T-shaped top-gate process and introduced it to CNT RF devices for the first time. Using a solution-based method to prepare a network CNT film on a quartz substrate, they achieved de-embedded *f*_T_/*f*_MAX_ of 23 GHz/10 GHz at a 140 nm gate length. They also constructed a Class A power amplifier, which demonstrated a power gain of 6 dB and a 1 dB compression point of 11.9 dBm at 500 MHz [56]. In 2016, Yu Cao et al. used CVD-grown CNT arrays and a dose-controlled floating evaporative self-assembly (DFES) method to achieve a CNT density of 40 tubes/μm on a quartz substrate. By shrinking the channel to 100 nm and incorporating the T-shaped gate, they realized de-embedded *f*_T_/*f*_MAX_ of 80 GHz/70 GHz and intrinsic *f*_T_/*f*_MAX_ of 100 GHz/70 GHz [61]. In 2019, Christopher Rutherglen et al. fabricated over 4700 RF transistors on a 100 mm wafer. By further improving CNT material preparation and device structure, they raised the semiconductor purity to 99.6% and the density to 40–60 tubes/μm, achieving extrinsic *f*_MAX_ and *f*_T_ both exceeding 100 GHz. They also conducted the first dual-tone test at 1.5 GHz, yielding an OIP3 of 17.6 dBm and a 1 dB compression point of −4.2 dBm, demonstrating the potential of CNT RF transistors in power amplification and linearity [62]. In 2024, Liu and Zipeng Pan reduced interface states and adopted a T-shaped gate structure, achieving extrinsic *f*_T_/*f*_MAX_ of 302 GHz/201 GHz (see Figure 5d) at a 50 nm gate length and *V*_ds_ = −1 V, far surpassing silicon-based MOSFETs of the same gate length [43].

#### 4.2.3. U-Shaped and Y-Shaped Gate Structures

Shown in Figure 5e is a U-shaped RF transistor. In a U-shaped gate structure, the gate stack curves upward at both ends, forming a ‘U’ shape. Here, the source and drain electrodes are vertically separated from the top gate metal (e.g., Ti/Sc) through a vacuum space, rather than overlapping as in traditional planar designs. The introduction of air gaps between the gate electrode and the source/drain contacts improves the electric field distribution within the channel, effectively reducing the gate-to-source (*C*_gs_) and gate-to-drain (*C*_gd_) parasitic capacitances, thereby increasing the device’s upper operating frequency limit. In 2011, Li Ding et al. from Peking University utilized a dual-layer PMMA mutual dissolution exposure technique to develop the U-shaped gate technology and applied it to a single CNT RF transistor. This work marked the first direct frequency-domain measurement verifying a *f*_T_ of 800 MHz for a single CNT FET [57]. In 2023, Zhou et al. from the same team introduced a Y-shaped gate to optimize the balance between gate resistance and parasitic capacitance. This enabled them to push the cutoff frequency of MOSFETs based on A-CNTs into the THz range for the first time. Their 35 nm gate-length device achieved a record extrinsic *f*_MAX_ of 1024 GHz and *f*_T_ of 551 GHz, with intrinsic *f*_MAX_/*f*_T_ reaching 1142 GHz/739 GHz, surpassing all previously reported MOSFETs. They also demonstrated a 30 GHz millimeter-wave amplifier based on A-CNTs with a gain of 21.4 dB, validating its potential for 6G communication systems-on-chip [63]. However, the fabrication process for U-shaped and Y-shaped gates is more complex than that of planar gates, requiring higher precision in etching and deposition.

While exceptional intrinsic cutoff frequencies only indicate a material’s speed ceiling and do not necessarily translate fully into practical device performance, candidate materials for the terahertz range are exceedingly rare, making CNTs highly promising. Such advancements could unlock their potential in applications like power amplifiers, high-linearity analog circuits, and digital/analog hybrid systems.

#### 4.2.4. Principle Demonstrations of RF Transistor Applications

Early efforts also included principle demonstrations of RF transistor applications using planar gate structures. In 2010, Zhenxing Wang from Peking University fabricated a top-gate CNT ambipolar transistor with Schottky contacts using a single small-bandgap CNT. By applying a 1 kHz AC signal to the gate, they achieved a 2 kHz output signal with over 0.15 dB gain and 95% spectral purity [64]. In 2012, through studying the transfer characteristics of ambipolar CNT transistors, the team adjusted gate bias to operate the device in three distinct regions: at zero bias, it functioned in the ambipolar region as an efficient frequency multiplier; with positive or negative gate bias, it operated in forward or reverse linear amplification regions due to pronounced saturation characteristics, enabling a voltage amplifier with a gain of 2. This unique ability to perform three functions in a single device stems from the bandgap-diameter-dependent ambipolarity of CNTs, providing a proof-of-concept for RF applications [65]. In 2013, the team developed an ambipolar FET based on CNT arrays, employing innovative ambipolar modulation techniques to achieve RF circuit performance up to 40 GHz. Their frequency multiplier produced a 40 GHz output from a 20 GHz input, with output power exceeding the noise floor by 10 dBm, surpassing traditional carbon-based RF circuit frequency and power limits and paving new paths for high-frequency carbon-based electronics [66].

In addition to individual RF transistors, efforts in RF-integrated systems have demonstrated the potential of CNT-based RF/digital hybrid circuits. In 2019, Liu Lijun from Peking University utilized high-performance CNT CMOS devices to integrate a voltage-controlled oscillator temperature sensor, carbon-based MOS circuits, a lithium-ion battery, and an antenna onto a flexible substrate. This showcased a complete IoT (Internet of Things) node system with sensing, signal processing, wireless transmission, and power supply capabilities. The system exhibited ultra-high energy efficiency, ultra-low dynamic power consumption, and a tunable frequency range of 0.4–1.5 GHz, covering the frequency bands required for NB (Narrow Band) IoT or GSM applications. This demonstrated the application potential of carbon-based digital/analog hybrid integrated systems in IoT [67].

#### 4.2.5. RF Diodes

RF diodes, with their advantages of fast high-frequency response, low junction capacitance, and low power consumption, play a critical role in high-frequency electronic systems such as terahertz communication, radar mixing, and 5G RF front-ends. As a significant development direction for RF diodes, CNT-based diodes have garnered considerable attention in recent years for terahertz-band device research [68].

In 2005, Harish M. Manohara and colleagues first demonstrated a Schottky barrier diode (SBD) based on single-walled carbon nanotubes, predicting a cutoff frequency (*f*_C_) in the THz range [69]. In 2011, Biswas et al. introduced the first p-n junction diode based on a chemically doped SWCNT random network, functioning as a half-wave rectifier with an operating frequency of 10 MHz. This outperformed the distortion characteristics of commercial silicon-based diodes at high frequencies during that period [70]. In 2016, Amanpreet Kaur and colleagues pioneered a p-n junction diode on a high-frequency-compatible flexible substrate (PEEK) using random network semiconducting SWCNTs, achieving microwave rectification at 18 GHz with a sensitivity of 4 V/W. However, the significant contact resistance in their Schottky diode limited the full potential of CNT materials [71]. In 2023, Chenqiao Xue from Peking University and colleagues reported a CNT network film-based diode with an intrinsic *f*_C_ exceeding 100 GHz, peaking at 170 GHz, and an extrinsic *f*_C_ surpassing 50 GHz, breaking the previous CNT diode record of 18 GHz. The diode exhibited a measured bandwidth of at least 50 GHz, enabling operation in the millimeter-wave band (30–300 GHz) and meeting the demands of 5G/6G communication and flexible electronics [72]. In the same year, Zhen Zhang et al. from Peking University reported a lateral-structure SBD based on high-density, high-purity aligned carbon nanotube arrays (A-CNTs). This device achieved a rectification current density of −0.78 mA/μm and a short-circuit current responsivity of 6.8 A/W. At zero bias, it exhibited an ultra-low junction capacitance of 2.5 fF and an extremely low series resistance of approximately 30 Ω, yielding an intrinsic *f*_C_ of up to 840 GHz. As shown in Figure 5f, this performance surpasses previously reported CNT-based and other emerging material SBDs, positioning it as an outstanding candidate for THz applications [58].

### 4.3. CNT Logic Circuits

#### 4.3.1. Development of CNT-Based Digital Integrated Circuits

The development of CNT-based logic circuits began with single-tube devices. In 2001, Bachtold et al. first demonstrated N-type and P-type ‘doping’ on a single CNT through gate modulation, fabricating multiple logic gate circuits with a handful of transistors, including inverters, NOR gates, flip-flop memory cells (SRAM), and ring oscillators, thus proving CNTs’ feasibility in digital circuits [73]. In 2007, Zhiyong Zhang et al. combined Pd-contacted p-type FETs and Sc-contacted n-type FETs on a single SWCNT, first achieving a doping-free CMOS process and successfully demonstrating an inverter [12]. In 2008, Qing Cao et al. built an integrated digital circuit on a flexible plastic substrate with nearly 100 transistors, including enhancement- and depletion-mode transistors, logic gates (inverters, NOR gates, NAND gates), and a 4-bit row/column decoder [74]. In 2016, Bingyan Chen et al. first realized a 4-bit adder and a 2-bit multiplier based on a CNT network, with all circuits operating under a single low voltage of 2 V [75].

After realizing small-scale logic circuits based on CNTs, researchers began to study larger-scale digital integrated circuits. In 2013, Shulaker et al. utilized highly aligned carbon nanotube arrays grown via CVD to demonstrate the world’s first computer entirely constructed from CNT field-effect transistors, marking a pivotal milestone in carbon-based electronics. This CNT computer comprised 178 CNT FETs, each containing 10–200 CNTs, and operated at a clock frequency of 1 kHz [76]. Limited by the material and process, its circuit performance and system integrity were relatively low. In 2014, they advanced this approach by fabricating ultra-large-scale CNT digital integrated circuits, demonstrating an inverter operating at 1 MHz and scaling channel dimensions below 20 nm, approaching advanced silicon technology nodes [77].

In 2017, Yingjun Yang et al. from Peking University first utilized doping-free CMOS technology to construct diverse logic and sequential circuits, achieving medium-scale integration. The circuits included a 2-to-1 multiplexer (MUX2_1), D-latch, T flip-flop, 1-bit full adder, and 4-bit adder. The 4-bit adder, consisting of 132 CMOS FETs, achieved a 100% yield, demonstrating the potential of this technology for medium-scale circuits [78]. Subsequently, Li Xiang from the same team developed CNT TFTs based on random network CNT films on flexible/degradable substrates, achieving ultra-low static power consumption (2.5 × 10^−12^ W/inverter). Under a single power supply of 2 V, they realized a full adder (13 logic gates) and read-only memory (ROM) with rail-to-rail output, demonstrating deep logic depth and high integration [79]. Subsequently, Li Xiang et al. utilized a random network carbon nanotube thin film to develop CNT thin-film transistors on flexible and degradable substrates. These devices achieved an extremely low static power consumption (2.5 × 10^−13^ W/inverter). Operating at 2 V, they also realized a full adder (13 logic gates) and a read-only memory (ROM) with rail-to-rail output, demonstrating deep logic depth and high integration [79].

**Figure 6:**
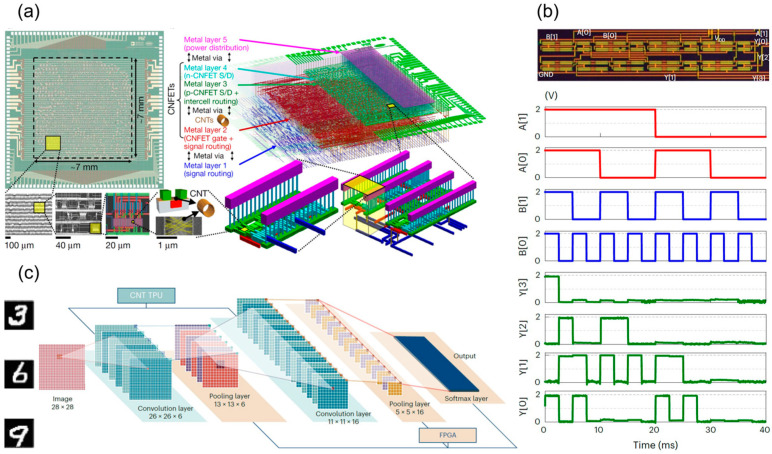
CNT-based integrated circuits. (**a**) Illustration of the largest CNT-based microprocessor comprising over 14,000 complementary CNT transistors [80]. Reproduced with the permission from SNCSC. (**b**) Optical image and output waveforms of 2-bit multiplier within a CNT-based tensor processing unit. Reproduced with permission from [81]; published by Springer Nature, 2024. (**c**) Illustration of a multilayer convolutional neural networks for MNIST image recognition including CNT TPUs and FPGAs. Reproduced with permission from [81]; published by Springer Nature, 2024.

In 2019, the Shulaker team employed solution-deposited CNT network films and overcame inherent CNT defects using techniques like RINSE, MIXED, and DREAM. They unveiled RV16X-NANO, see Figure 6a, the world’s first 16-bit general-purpose CNT microprocessor based on the RISC-V instruction set [80]. Capable of executing standard 32-bit instructions and processing 16-bit data and addresses, it performed operations including instruction fetch, decode, register access, computation, and data storage. Comprising over 14,000 complementary CMOS CNT FETs, it was fabricated using industry-standard design flows and processes, further validating CNT technology’s potential to surpass silicon-based electronic systems. Projections suggest that CNT FET-based digital systems could achieve an order-of-magnitude improvement in energy efficiency over silicon technologies. Due to its poor device performance, its operating frequency is only 10 kHz, and the number of transistors is only 57,600. There is still a significant gap in circuit integration scale and performance compared with the 80,386 chip released by Intel in 1985 [80].

Building on prior circuit research, Jia Si et al. reported in 2024 the first tensor processing unit (TPU) based on 3000 CNT field-effect transistors. Employing a systolic array architecture, this TPU supported parallel 2-bit integer multiply-accumulate (MAC, see Figure 6b) operations for convolution and matrix multiplication. A five-layer convolutional neural network (CNN) built on this TPU achieved an 88% accuracy rate in the MNIST handwritten digit recognition task with a power consumption of just 295 μW, showcasing ultra-low-power computing capabilities. The schematic of CNN is shown in Figure 6c. Simulations suggest that an 8-bit CNT TPU based on a 180 nm process could achieve a clock frequency of 850 MHz and an energy efficiency of 1 TOPS/W (1 tera-operations per second per watt), surpassing traditional silicon-based technology [81].

#### 4.3.2. Speed of High-Performance Digital Integrated Circuits

**Figure 7:**
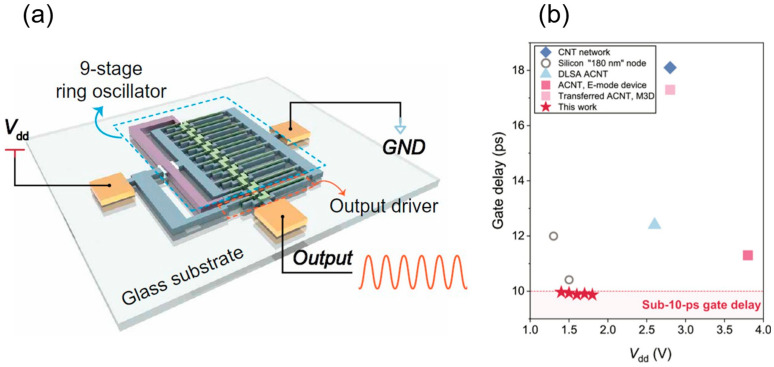
(**a**) Illustration of 9-stage ring oscillator and (**b**) comparison of the gate delay versus supply voltage between published ring oscillators on CNTs reported by Xiaohan Cheng. From [42]. Reprinted with permission from AAAS.

Early devices were constrained by material purity and performance, with gate delays on the order of microseconds. In 2006, the IBM team optimized fabrication processes to construct a CMOS five-stage ring oscillator on a single CNT, achieving an oscillation frequency of 52 MHz and reducing single-stage gate delay to 1.9 ns, marking the first demonstration of CNTs’ high-speed potential [82]. In 2017, Yang et al. from Peking University built a five-stage ring oscillator on a CNT network film using ambipolar devices, achieving a 17.4 MHz oscillation frequency and a 5.6 ns single-stage gate delay [83]. In that same year, Han et al. from IBM developed a CMOS five-stage ring oscillator on A-CNTs with a 100 nm gate length, reaching a peak oscillation frequency of 282 MHz and a single-stage gate delay of 355 ps, greatly surpassing previous records for the speed of carbon-based digital circuit [84]. Meanwhile, Zhong et al. from Peking University, through extensive device structure and process optimization, utilized a CNT network film and a 115 nm gate-length air-gap PMOS to achieve a five-stage ring oscillator with an oscillation frequency up to 5.54 GHz and a single-stage gate delay of just 18 ps, representing the best among low-dimensional material circuits at the time [85]. In 2020, leveraging self-assembled CNT arrays via the DLSA method, Liu et al. from Peking University fabricated a PMOS five-stage ring oscillator with a 165 nm gate length, achieving a peak oscillation frequency of 8.06 GHz and a single-stage gate delay of 12.4 ps [13]. Operating at a lower voltage, this outperformed commercial silicon devices of the same size, fully demonstrating CNTs’ potential for high-performance digital circuit applications. In 2021, Yanxia Lin et al. from Peking University further refined device structure and process, realizing enhancement-mode transistors and multi-stage ring oscillators with a single-stage gate delay of 11.3 ps [23]. In 2024, Xiaohan Cheng et al. introduced low-loss glass wafers as substrates for A-CNT FETs for the first time, reducing parasitic effects and power consumption. As shown in Figure 7a,b, their 9-stage ring oscillator achieved a gate delay of 9.86 ps (*V*_DD_ = 1.8 V, frequency = 5.63 GHz), continually pushing transistor speed limits [42]. As CNT materials and device fabrication techniques continue to advance, their high-speed, high-performance potential in digital computing circuits will be further explored and validated.

### 4.4. Breakthroughs and Applications of CNT 3D Integration Technology

#### 4.4.1. Evolution of Monolithic 3D Integration

**Figure 8:**
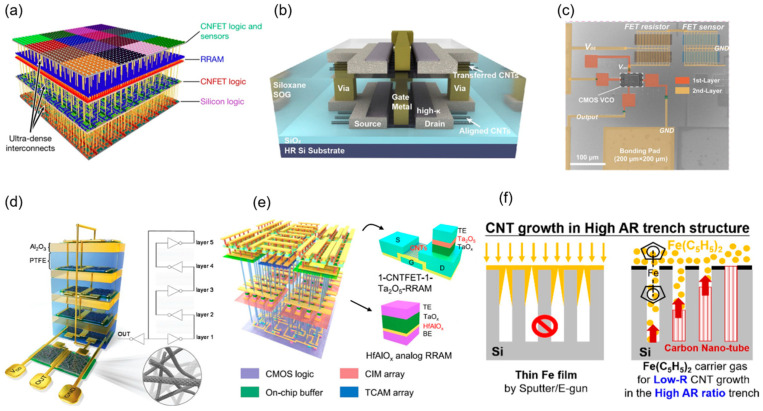
(**a**) Three-dimensional integration for computing, data storage, and sensing on a single chip [87]. Reproduced with the permission from SNCSC. (**b**) Detailed architecture of stacked A-CNT FETs for high-performance integrated circuits. Used with permission of John Wiley & Sons—Books, from [89]; permission conveyed through Copyright Clearance Center, Inc. (**c**) Microscope image of the M3D H_2_ sensing system including CNT logic transistors and sensors. Reprinted with permission from [90]. Copyright 2011 American Chemical Society. (**d**) Schematic of the laminated 3D 5-stage ring oscillator showing CNT films as the channels and PTFE/Al_2_O_3_ as the separators. VDD, OUT, and GND represent the supplied voltage, output signal, and ground of the circuit, respectively. Used with permission of Royal Society of Chemistry, from [91]; permission conveyed through Copyright Clearance Center, Inc. (**e**) Architecture of the M3D-LIME chip. Reproduced with permission from [92]. Published by Springer Nature, 2023. (**f**) The growth mechanism of CNT in trench structure with high aspect ratio [93]. Reprinted with permission from IEEE Proceedings.

Early research focused on validating the performance of single-layer carbon nanotube devices. However, with the maturation of CNT purification techniques and A-CNT assembly methods (e.g., DLSA), 3D integration has transitioned from concept to practice. The low-temperature process compatibility of CNTs (<300 °C) provides an ideal platform for monolithic 3D integration. In 2014, Shulaker team from Stanford University demonstrated the first heterogeneous 3D integrated system with a four-layer structure; see Figure 8a [86]. The bottom layer consisted of silicon-based logic circuits, the middle two layers were RRAM, and the top layer featured CNT logic circuits, offering an initial exploration of the vertical integration feasibility of carbon-based and silicon-based technologies. In 2017, the team vertically stacked computing, memory, and sensing functions on a single chip, achieving a fine-grained, high-density monolithic 3D integration architecture [87]. Here, CNT FETs were fabricated at temperatures below 200 °C, compatible with traditional silicon CMOS processes. The energy-delay product (EDP) of these CNT FETs was an order of magnitude lower than that of silicon MOSFETs, enhancing energy efficiency. The interconnect process utilized metal interlayer vias with a 2 μm pitch, enabling over 1 million vertical connections, which is hundreds of times denser than traditional chip-stacking technologies with TSV pitches of 10–40 μm. A prototype demonstrated environmental gas sensing and classification, highlighting its potential for applications like environmental monitoring. In 2018, the team further optimized the process, showcasing the efficiency and robustness of the monolithic 3D integrated system in language classification tasks, achieving a 35-fold energy efficiency improvement and a threefold reduction in area footprint during training and inference tasks [88]. However, these CNT devices exhibited relatively low performance and slow circuit speed.

In 2019, Yunong Xie et al. from Peking University optimized the 3D integration process, producing CNT FETs with exceptional performance, including a high drive current (77 μA/μm), high transconductance (111 μS/μm), and low subthreshold swing (180 mV/decade) [94]. They successfully fabricated a 3D five-stage ring oscillator with an oscillation frequency of up to 680 MHz and a stage delay of 0.15 ns, representing the highest speed achieved for 3D CNT-based integrated circuits at that time. In 2023, Chenwei Fan et al. from the same team proposed an M3D integration technology based on aligned carbon nanotube arrays, successfully enabling the stacking of multilayer high-performance CNT FETs and ICs; see Figure 8b for M3D structure [89]. They employed a low-temperature process (maximum: 220 °C) to prepare spin-on glass (SOG) with a low dielectric constant (~3) as the interlayer dielectric (ILD), effectively reducing interlayer parasitic capacitance and providing an excellent planarized surface for upper-layer device fabrication. The upper-layer A-CNT films, prepared via a clean transfer process, exhibited a carrier mobility of up to 650 cm^2^·V^−1^·s^−1^, enabling upper-layer CNT FETs to achieve a high drive current (1 mA·μm^−1^) and peak transconductance (0.98 mS·μm^−1^) under low-temperature conditions. The lower-layer A-CNT FETs, despite undergoing ILD growth and upper-layer processing, retained excellent performance with a drive current of 1.84 mA·μm^−1^ and transconductance of 1.65 mS·μm^−1^. They further demonstrated a five-stage ring oscillator (RO) based on the M3D architecture, achieving a gate propagation delay of 17 ps within an active area of just ~100 μm^2^, which represents the fastest and most compact M3D IC reported to date. Compared to silicon CMOS and other emerging materials, this technology showed significant advantages in drive current and transconductance. In the same year, they also demonstrated an M3D integration using network CNT films, integrating CNT CMOS circuits on the bottom layer and a CNT hydrogen sensor array on the top layer (see Figure 8c), connected with interlayer vias [90]. This system converted hydrogen concentrations (8–128 ppm) into digital frequency signals (0.78–1.11 GHz) with a sensitivity of 2.75 MHz/ppm. The response time and recovery time of the sensor were 197 s and 289 s, respectively, suitable for real-time monitoring. Additionally, the system supported a low-power mode (frequency range of 0.41–0.62 GHz at *V*_DD_ = 3 V), making it viable for Internet of Things (IoT) applications. This integration approach offers a novel architecture for smart sensing chips, enabling simultaneous sensing and signal processing.

In terms of vertical integration, Yang Jian et al. successfully constructed a 3D inverter and a 5-stage ring oscillator by layer-by-layer stacking of CNT TFTs and PTFE/Al_2_O_3_ separators (see Figure 8d). The 3D oscillator achieved an approximately 80% reduction in area compared to its planar counterpart. However, its oscillation frequency was limited by parasitic capacitance and resistance [91]. In addition, Yijun Li et al. developed a monolithic 3D hybrid memory architecture chip (named M3D-LIME). Specifically, CNTFETs were utilized in the third layer to construct a ternary content-addressable memory array based on Ta_2_O_5_ resistive random-access memory (RRAM) for template storage and matching, enabling one-shot learning. The chip achieved a 96% classification accuracy on the Omniglot dataset, with an energy efficiency 18.3 times higher than that of GPUs and a speed 2.73 times faster than 2D chips [92]. In the future, by optimizing the manufacturing process of CNT FETs and further reducing the thermal budget, CNT 3D integration is expected to promote the realization of more efficient and compact neuromorphic computing and data-intensive applications.

#### 4.4.2. Potential of Carbon Nanotubes in Thermal Optimization of 3D Integrated Circuits

Three-dimensional integration offers significant energy efficiency advantages for high-performance circuits based on CNTs. However, the thermal management challenges arising from high-density integration represent a critical bottleneck to realizing its full potential. In practical applications, the thermal conductivity of CNT arrays or network films is significantly lower than that of individual CNTs, primarily due to substantial thermal resistance caused by inter-tube contacts forming localized hotspots and inconsistent alignment directions. Therefore, at the material level, it is essential to fabricate high-quality, highly aligned CNT arrays to preserve their intrinsic high thermal conductivity and prevent the formation of localized hotspots. To enhance inter-tube thermal coupling, current strategies include polymer filling and substrate processing [95], which improve the overall thermal conductivity of the structure.

In terms of interconnection, traditional copper (Cu) interconnect technology faces multiple challenges that severely limit further improvements in device performance and reliability. First, in high-aspect-ratio (AR > 25) structures, Cu interconnects exhibit a significant increase in resistivity, resulting in greater signal delay and power consumption, which struggles to meet the demands of high-performance computing. Second, Cu’s limited thermal conductivity fails to effectively dissipate the heat accumulated in 3D ICs, causing elevated device temperatures that degrade performance and long-term reliability. Additionally, Cu’s low Young’s modulus makes it inadequate to withstand the mechanical stresses of high-density integration, while its thermal expansion coefficient (CTE) of 16.5 mismatches that of silicon, exacerbating thermal stress and compromising structural stability. Finally, the compatibility of traditional Cu interconnect fabrication with back-end-of-line processes is poor, particularly in high aspect ratio structures, hindering the realization of high-quality interconnects and restricting further miniaturization and integration of 3D ICs. These compounded issues underscore the urgent need for novel interconnect materials.

Metallic carbon nanotubes emerge as a promising interconnect material, offering a highly promising solution to address the critical challenges in high-density 3D ICs. In 2020, P.-Y. Lu from National Taiwan University utilized a novel gas-phase reactant, Fe(C_5_H_5_)_2_, to synthesize CNTs, demonstrating its advantages as a material for high aspect ratio (>25) through-silicon vias [93]. The growth mechanism of CNTs to occupy the high AR trench is shown in Figure 8d. Their measurements revealed an exceptionally low resistivity (~10^−6^ Ω·m), reducing signal delay and power consumption to meet high-performance computing requirements. Moreover, CNTs possess a thermal conductivity of ~800 W·m^−1^·K^−1^, twice that of Cu, enabling effective heat dissipation, lowering device temperatures, and enhancing both performance and reliability. Additionally, CNTs exhibit a Young’s modulus of ~1000 GPa, far exceeding that of Cu (117 GPa) and Si (150 GPa), allowing them to endure the mechanical stresses of high-density integration. Their CTE, nearly zero or even negative (~−2 × 10^−6^ K^−1^), closely matches silicon’s thermal expansion properties, substantially reducing thermal stress and bolstering structural stability. Their demonstration of CNTs as vertical interconnects lowered device temperature by ~15 °C, reduced keep-out zone area by ~80%, and improved system reliability by approximately tenfold, highlighting the electrical reliability and layout advantages of metallic CNTs as vias materials in 3D ICs [93]. Furthermore, other high thermal conductivity materials such as graphene or even metallic carbon nanotubes can be used as intra-layer interconnects to achieve all-carbon circuits and further quickly transfer heat to external heat sinks [96]. On the substrate side, integrating CNTs with high thermal conductivity materials like diamond or BN provides an effective thermal extraction path. Further improvements can be achieved by incorporating thermal optimization strategies, including graded thermal vias with spatially optimized TSV distributions, increased TSV density in high-temperature zones, and embedded cooling channels such as microfluidic or diamond-based channels, which efficiently dissipate hotspots and reduce thermal accumulation in 3D systems.

Based on the above advantages and strategies, local hotspots and thermal accumulation can be significantly reduced, while system stability and scalability are greatly enhanced, laying a solid foundation for thermal management in future high-density, low-power CNT-based 3D integrated electronic systems.

### 4.5. CNT-Based Neuromorphic Devices

The conventional von Neumann architecture faces significant challenges, including the ‘memory wall’ and energy consumption bottlenecks, particularly in applications requiring efficient, low-power data processing, such as edge computing and the Internet of Things (IoT). In contrast, the human brain, with its highly parallel architecture (~10^11^ neurons and ~10^15^ synaptic interconnections), achieves remarkable information processing efficiency with a power consumption of approximately 20 W [97]. Consequently, developing electronic devices that emulate biological synaptic functions has become a critical step toward constructing neuromorphic systems based on non-von Neumann architectures. These systems aim to replicate the efficient information processing capabilities of biological neural networks at the hardware level, thereby overcoming the inherent limitations of traditional computing architectures in terms of energy efficiency and parallelism. Ideal synaptic devices should exhibit analog programmability, high dynamic range, low power consumption, long-term memory retention, and robustness. CNTs, owing to their exceptional electrical properties and high sensitivity to charge trapping at interfaces or within dielectric layers, are promising materials for constructing synaptic transistors [98,99,100]. Recent research has not only explored the charge-trapping mechanisms and fabrication of CNT-based synaptic devices [101,102,103] but also validated their potential in applications such as biomimetic sensing [104], flexible electronics [100,105,106], and autonomous driving [107].

**Figure 9:**
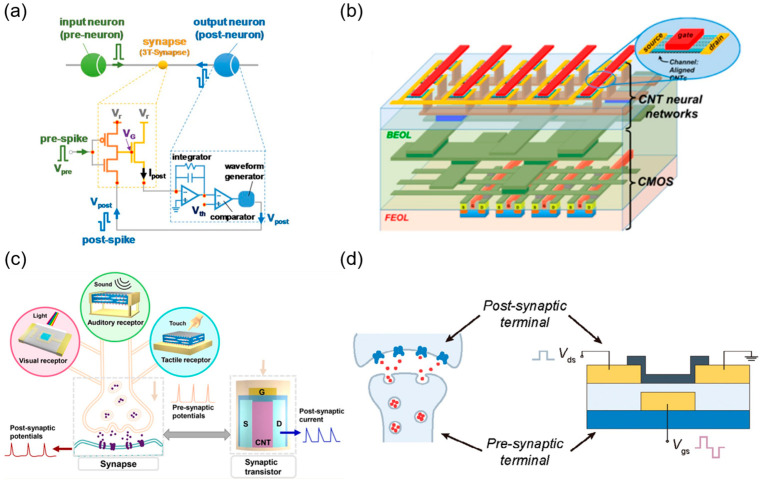
CNT neuromorphic devices. (**a**) Neuron circuit with each synapse. The neuron circuit is composed of a leaky integrator, comparator, and waveform generator. Reprinted with permission from [100]. Copyright 2017 American Chemical Society. (**b**) Conceptual back-end-of-line (BEOL) integration of aligned CNT FETs for artificial neural network implementation in crossbar configuration. Reprinted with permission from [108]. Copyright 2017 American Chemical Society. (**c**) Schematic illustration of artificial sensory-memory system consisting of corresponding biomimetic physical sensors and artificial synaptic transistor. Reprinted with permission from [104]. Copyright 2021 American Chemical Society. (**d**) Schematic biological synapse, and the pre- and post-synaptic stimulation of the memory devices for synaptic operation. Reproduced with permission from [107]; published by John Wiley and Sons, 2024.

In 2017, Sungho Kim et al. proposed a three-transistor synaptic device (3T-Synapse) based on networked CNTs, incorporating an Au floating gate to enable an adjustable weight update protocol, which significantly enhanced analog conductance modulation (total variation margin ΔG up to 57.5) [100]. Their simplified spike-timing-dependent plasticity (STDP) scheme reduced peripheral circuit complexity (Figure 9a), and system-level simulations demonstrated the device’s performance in MNIST digit recognition tasks (recognition accuracy of 60–70%) [100]. In 2018, Ivan Sanchez Esqueda et al. developed synaptic transistors using wafer-scale, highly aligned CNT arrays (purity > 99.9%, density > 60 tubes/μm) (Figure 9b), leveraging T-shaped gate structures and charge trapping in high-k HfO_2_ dielectrics to achieve synaptic behavior [108]. These devices exhibited over an order of magnitude conductance modulation, high uniformity (hysteresis window ~0.9 V, stable over 1000 cycles), and optimized learning performance, achieving high recognition accuracy with low training iterations in MNIST handwritten digit recognition tasks [108]. In 2021, Wan et al. integrated phototransistors and ferroelectric nanogenerators (FENG, for auditory and tactile perception) with CNT-based synaptic transistors on flexible substrates, see Figure 9c, successfully emulating three human sensory modalities (visual, auditory, and tactile) and synaptic memory behaviors. Their system demonstrated the Atkinson–Shiffrin multistore memory model and Pavlov’s classical conditioning experiment, validating short-term to long-term memory transitions and associative learning capabilities [104]. In 2024, Pei et al. fabricated synaptic transistor (view Figure 9d for device schematic) memories with a high switching ratio (>10^5^), large memory window (12 V), low response delays (tens of nanoseconds), and ultralow energy consumption (2.16 fJ per weight programming) [107]. They designed and implemented a 3 × 3 hardware convolution kernel based on nine transistors, enabling parallel image processing at 1 Mbit/s channel rates, with successful validation in Sobel edge detection and video processing applications, achieving performance comparable to state-of-the-art memristor and transistor-based solutions [107]. These advancements highlight the transformative potential of CNT-based synaptic devices, paving the way for scalable, energy-efficient neuromorphic systems capable of addressing complex real-world computing challenges.

### 4.6. CNT Optoelectronic Integration

In the post-Moore era, traditional semiconductor technologies, constrained by the slowdown of Moore’s Law, face physical limits in bandwidth, power consumption, and latency, making it challenging to meet the performance demands of future high-speed communications, artificial intelligence, and quantum computing. To overcome data transmission bottlenecks and energy efficiency limitations, on-chip optical communication has emerged as a critical solution, driving the rapid development of optoelectronic integration circuit (OEIC) technologies. Recent breakthroughs in silicon photonics, III-V semiconductor integration, and heterogeneous material bonding have significantly promoted the commercialization of OEIC chips in applications such as data center interconnects, 5G/6G communications, LiDAR, and biosensing [109]. For instance, silicon photonic modules have partially replaced traditional discrete devices. However, conventional OEIC relies on multiple materials (e.g., III-V, Ge, Si), leading to manufacturing conflicts between photonics and electronics, which hinders high-density integration [110]. CNTs, with their excellent optoelectronic properties and compatibility with silicon-based materials, have emerged as a highly promising alternative for OEIC [111]. Researchers have achieved significant progress in CNT-based optoelectronic devices by integrating CNT electrical devices with optical structures such as waveguides and photonic crystals, developing on-chip light-emitting devices [112,113], modulators [114], detectors [115,116,117,118], and optoelectronic quantum integration [119], with continuous performance improvements through structural optimization.

**Figure 10:**
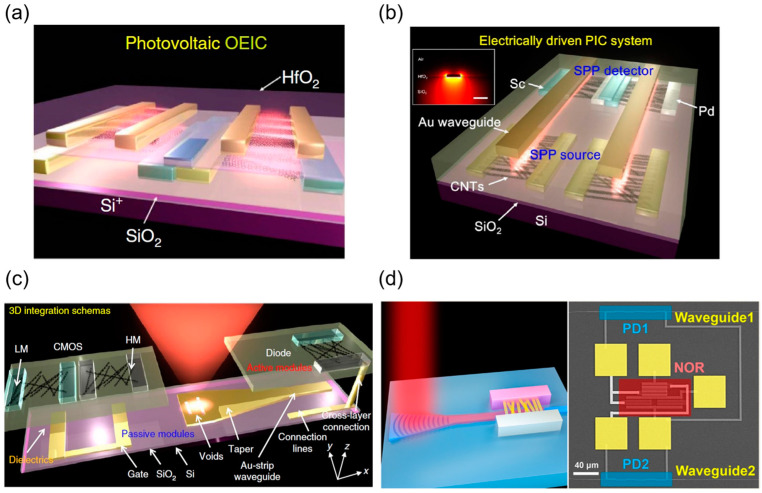
CNT optoelectronic integration. (**a**) Schematic of the vertical near-field OEIC, consisting of a top-layer emitter and a bottom-layer cascading detector. Reproduced with permission from [120]; published by Springer Nature, 2017. (**b**) Electrically driven PIC system based on CNTs. From [121]. Reprinted with permission from AAAS. (**c**) Schematic of the 3D integration of plasmonics and electronics [110], reproduced with permission from SNCSC. (**d**) Schematic diagram showing the structure of a waveguide-integrated photodiode and CNT OEIS for WDM system. Reprinted with permission from [122]. Copyright 2020 American Chemical Society.

In terms of advancing device integration, researchers have conducted multiple proof-of-concept demonstrations for on-chip OEIC. In 2017, Yang Liu et al. pioneered the use of CNTs as a single material to achieve 3D monolithic OEIC (Figure 10a) using CMOS-compatible, low-temperature, doping-free processes, integrating photovoltaic detectors, electrically driven emitters, and signal processing circuits [120]. Through a 2 × 2 array, they validated interlayer parallel optical communication using channel-division multiplexing mapped data, demonstrating the potential for high-bandwidth communication between microprocessors and memory with theoretical rates exceeding 10 Gbps [120]. They also developed heterogeneous optoelectronic logic gates (e.g., AND gates) with optical and electrical isolation to prevent crosstalk during signal transmission [120]. In the same year, they reported the first CNT-based deep subwavelength (feature sizes ~λ/7 to λ/95, λ = 1340 nm) plasmonic interconnect circuit (PIC, see Figure 10b), integrating an electrically driven surface plasmon polariton (SPP) source, photovoltaic cascaded detectors, and Au strip waveguides, overcoming the size and material limitations of traditional optoelectronics [121]. Furthermore, as shown in Figure 10c, they achieved vertical integration of passive plasmonic components with active electronic devices, utilizing waveguide-fed slot antennas (WFSAs) to construct 3D OEIC, including unidirectional receivers, wavelength-polarization multiplexers, and CMOS signal-processing circuits [110]. In 2020, Ze Ma et al. achieved monolithic integration of silicon waveguides, CNT photodetectors, and CMOS logic gates, incorporating grating couplers, single-mode waveguides, multimode interferometers, and photodetectors with a responsivity of 12.5 mA/W (1530 nm) [122]. They further validated the compatibility of CNT OEIC with wavelength division multiplexing systems, processing multi-wavelength optical signals using two serpentine contact photodiodes and an NOR gate (view Figure 10d), demonstrating optical-to-electrical signal conversion and logic operations, thus providing a practical framework for on-chip optical interconnects and fiber-optic communications [122]. Future efforts should focus on preparing ultra-high-purity chirality-controlled CNTs, achieving high-density aligned arrays, improving device speed and optical coupling efficiency, and developing contamination-free integration processes with silicon platforms to enable high-speed, low-loss on-chip optoelectronic integration.

### 4.7. Devices of Novel Mechanism

#### 4.7.1. Dirac Source Transistor

Beyond pursuing high performance, the industry increasingly prioritizes reducing device power consumption, with lowering *V*_DD_ being the most effective method to decrease dynamic power. In early MOSFET scaling, *V*_DD_ progressively dropped from 12 V to 5 V, 3.3 V, and eventually 0.6 V. However, this scaling is constrained by the threshold voltage (*V*_th_) [123]. To maintain transistor performance, *V*_th_ cannot be reduced indefinitely, as it would increase leakage current. Due to the Boltzmann limit in classical MOSFETs, which stems from the thermal tail of carrier distribution, devices require at least 60 mV at room temperature to switch current by an order of magnitude in the subthreshold region, preventing further reductions in operating voltage. Thus, introducing new principles, materials, and structures to optimize dynamic power and achieve subthreshold swings below 60 mV/dec is critical.

**Figure 11:**
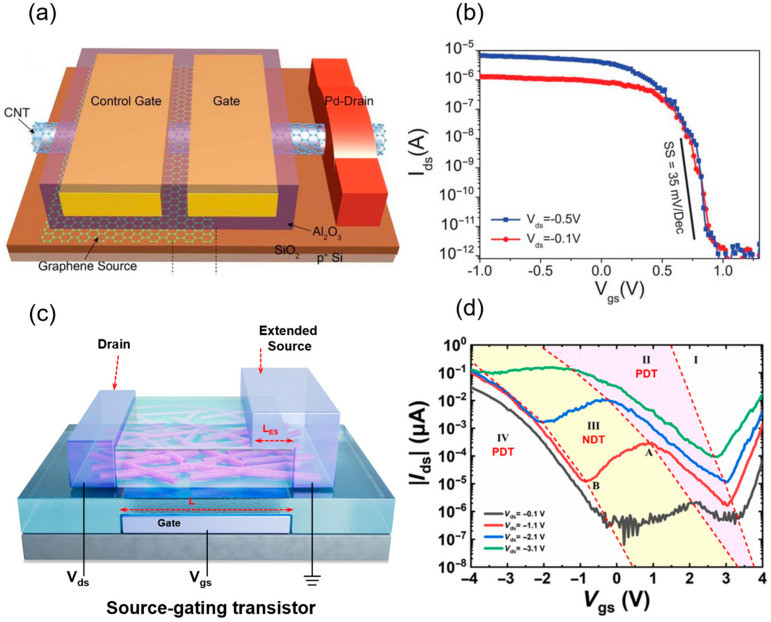
Novel-mechanism devices. (**a**) Schematic illustration and (**b**) transfer characteristics of Dirac-source FETs with lowest *SS* of 35 mV/dec. From [126]. Reprinted with permission from AAAS. (**c**) Illustration picture and (**d**) typical transfer characteristics of a source-gating transistor built for ternary logic. Reproduced with permission from [96]; published by The American Association for the Advancement of Science, 2025.

The two mainstream sub-60 mV/dec devices, including tunneling FETs (TFETs) and negative capacitance FETs (NCFETs), each have notable drawbacks: TFETs suffer from low on-state current and complex fabrication, while NCFETs lack a fully understood mechanism and rigorous performance validation [124,125]. In 2018, the team from Peking University proposed a novel sub-60 mV/dec device based on carbon nanotubes, which is a Dirac Source Transistor (DSFET) [126]. The specific device structure is in Figure 11a. Unlike conventional transistors, where the source-end density of states increases with rising Fermi level, limiting *SS* by thermal activation, this design employs an N-doped graphene/intrinsic graphene homojunction as the source contact for P-type devices. This reduces the density of states as the Fermi level rises, lowering *SS* at room temperature to an average of 40 mV/dec (as low as 35 mV/dec in some cases, see Figure 11b) without altering the carrier distribution function, thus breaking the Boltzmann limit. The approach leverages Klein tunneling in the graphene homojunction (with a transmission coefficient near 1) and a low-barrier, penetrative contact between graphene and CNTs for efficient carrier injection. Moreover, *V*_DD_ can be reduced to 0.5 V while maintaining a high on–off ratio (>10^5^) and delivering an *I*_on_ comparable to silicon 14 nm node FETs, with dynamic power consumption reduced to just one-third [126].

Despite their promise, DSFETs face challenges in practical application. The fabrication of graphene-CNT heterojunctions is complex, requiring precise interface quality control to minimize scattering centers. Additionally, CNT diameter distribution needs further optimization to ensure device uniformity. Future efforts must integrate advancements in material preparation, device structure, and circuit design to transition DSFETs from the lab to industrial applications.

#### 4.7.2. Ternary Logic Device

Traditional binary logic has dominated computing technology for over half a century. As silicon-based CMOS technology approaches its physical limits, quantum effects and interconnect power consumption have emerged as bottlenecks, exposing limitations in information density, power efficiency, and circuit complexity. To address this challenge, Xuehao Zhu et al. from Peking University proposed a CNT-based source-gate transistor (SGT) design; see Figure 11c [96]. By extending the source electrode into the channel to form a controllable p-n homojunction, they ingeniously achieved a ternary logic device with a negative differential transconductance (NDT) effect, as shown in Figure 11d. They successfully constructed high-performance circuits, including ternary inverters, NMIN/NMAX logic gates, a ternary 6T-SRAM, and an 8 × 8 ternary neural network (TNN), with the TNN achieving 100% accuracy in handwritten digit recognition tasks, representing the most advanced ternary circuit based on low-dimensional materials to date [96]. This research not only overcomes the information storage constraints of binary logic but also demonstrates the superior performance and large-scale integration potential of CNT-SGTs in multi-valued logic architectures, paving a new path for low-power, high-efficiency computing in the post-Moore era.

## 5. Technical Challenges and Development

### 5.1. CNT Materials

The realization of high-performance CNT devices heavily relies on breakthroughs in material preparation technologies, particularly the scalable production of ultra-high-purity semiconducting CNTs and the effective suppression of metallic CNTs. For high-performance CNT electronics, ultra-high-purity, high-density parallel CNT arrays represent the ideal material. In 2024, Yumeng Ze et al., from the perspective of integrated circuit applications, explicitly outlined material standards for A-CNTs across various technology nodes (e.g., 90 nm, 22 nm, 7 nm, and 3 nm), specifying distribution requirements for parameters such as length, diameter, density, and semiconducting purity [127]. Specifically, for the 3 nm node, ideal A-CNT materials should meet the following criteria: semiconducting purity > 99.9999% (‘six nines’); CNT length > 1 μm; diameter controlled at 1.3 nm ± 0.12 nm with chirality sorted; high-density alignment (>300 tubes/μm) with orientation < 7°; pitch variation < 0.8 nm; wafer-scale production; and high yield.

Currently, two primary strategies dominate the scalable preparation of high-purity, high-density semiconducting CNT arrays. The first involves selective catalytic growth of CNT arrays on substrates via CVD, followed by post-processing techniques, such as high-current burnout of metallic tubes, to enhance semiconducting purity. The second employs solution-based methods to post-process and purify pre-synthesized CNTs, followed by deposition, dip-coating, or self-assembly to form CNT arrays.

#### 5.1.1. Selective Catalytic Growth via CVD

The CVD method [66] offers precise control over carbon nanotube properties, such as diameter, length, type (metallic or semiconducting), and even chirality, while producing high-quality, well-aligned CNTs without the contamination or damage associated with solution-based assembly.

**Figure 12:**
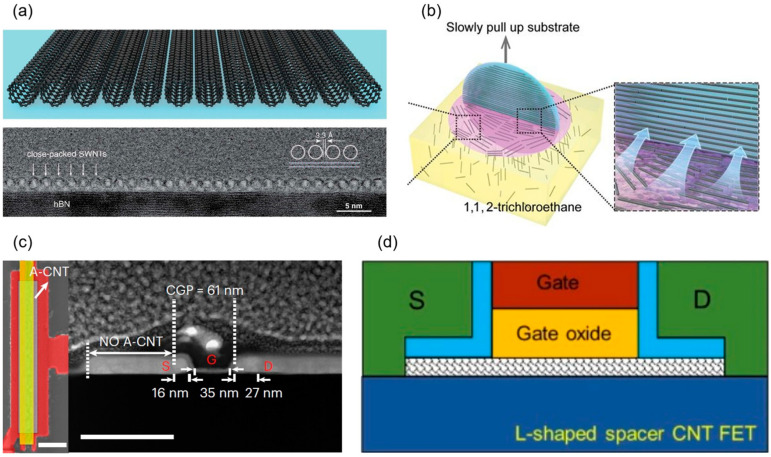
(**a**) A schematic of the close-packed uniformly spaced homochiral SWCNT array directly grown on hBN through chemical vapor deposition. From [44]. Reprinted with permission from AAAS. (**b**) Aligned CNT arrays prepared by DLSA method with tunable density and high semiconducting purity. From [13]. Reprinted with permission from AAAS. (**c**) False-color SEM image of three top-gated CNT FETs with a CGP of 175 nm superior to 45 nm silicon technology node transistors in terms of size and electronic performance. Reproduced with permission from [33]; published by Springer Nature, 2023. (**d**) Schematic picture of L-shaped-spacer CNT FET [131], reproduced with permission from SNCSC.

In terms of alignment, various research groups have developed CVD-based techniques, such as growing CNT arrays along specific substrate crystal orientations or leveraging buoyancy effects from stable gas flow density differences. These approaches achieve alignment precision below 0.01°, but typically result in lower densities [50,128]. For semiconducting purity, optimizing catalyst design can enhance the selective growth of chirality-enriched semiconducting CNTs. For instance, the Co_6_W_7_ catalyst boosts the growth selectivity of (12.6) chirality CNTs to 94% [129]. Regarding array density, the Jin Zhang group at Peking University, through specialized catalyst design, produced arrays with a density of 130 tubes/μm, though the diameter distribution remained broad (0.8–1.6 nm) [130]. In 2025, Zhichun Zhang et al. directly grew high-density, highly aligned, single-chirality (10.7) CNT arrays on an hBN substrate via CVD (see Figure 12a), achieving an inter-tube spacing of 0.33 nm, a mobility of 2000 cm^2^·V^−1^·s^−1^, and a maximum device current density of 6 mA/μm with an on–off ratio exceeding 10^7^. However, wafer-scale production was not realized [44].

In summary, CNT arrays prepared via CVD, even with post-processing, refs. [128,132], cannot simultaneously fulfill all requirements of high density, high purity, directional alignment, and wafer-scale large-area fabrication. They face challenges such as limited chirality control, difficulty in fully eliminating metallic CNTs (m-CNTs), catalyst agglomeration during high-density growth, inadequate uniformity, and elevated growth temperatures (typically > 800 °C). There are many influencing factors, such as the choice of catalyst type, effective control of parameters such as catalyst purity, size, and thermal stability, which have a significant impact on the purity, density, and uniformity of carbon nanotubes, and common problems such as catalyst deactivation, migration, and aggregation during the growth process need to be solved. This method also demands high requirements on the substrate lattice structure, ambient temperature, atmosphere, and gas flow rate during growth, and these processes still need to be further explored.

#### 5.1.2. Post-Processing Purification for CNT Array Preparation

The post-processing purification method is a two-step strategy. First, CNTs synthesized via arc discharge (AD) [133], laser ablation (LA) [134], or chemical vapor deposition [135] are purified using techniques such as aqueous two-phase extraction, density gradient ultracentrifugation, or column chromatography [136]. Subsequently, these purified CNTs are self-assembled onto a substrate. This approach facilitates effective large-scale preparation and performance uniformity. Arc discharge and laser ablation are high-yield methods that, compared to low-temperature processes like CVD, produce CNTs with higher quality and fewer structural defects, though controlling diameter and chirality remains challenging. For purification, the use of polycarbazole (PCz) dispersants through multiple dispersion and sorting steps can improve the purity of semiconducting CNTs (s-CNTs) to 99.99995% [13]. This method has been widely adopted in electronic device fabrication. However, the centrifugation and ultrasonic processes involved in the purification process can easily cause defects and breakage of CNTs. Therefore, while ensuring purity, it is necessary to minimize the impact of this process or reduce these processes.

The second step involves assembling the high-purity s-CNT solution into arrays on a target substrate for subsequent device and circuit fabrication. Assembly techniques are classified based on their principles: Langmuir film assembly, evaporation-driven self-assembly, template-assisted self-assembly, and DLSA.

The Langmuir method leverages surface tension to deposit a CNT suspension onto a water surface, forming a single-layer CNT film through a compression–retraction–compression cycle. This technique has achieved CNT arrays with densities of 50–100 tubes/μm or exceeding 500 tubes/μm, but density control remains challenging. Resulting devices either exhibit low current density or poor gate control, and no wafer-scale production has been reported [137,138].

The evaporation-driven self-assembly method involves immersing a substrate in a CNT solution, forming a liquid–solid–gas three-phase contact line, and slowly withdrawing the substrate. The coffee-ring-like effect from solvent evaporation at the contact line facilitates parallel CNT alignment on the substrate. It produces arrays with a density of ~50 tubes/μm and an orientation angle < 15°. However, the resulting CNT films are distributed in striped patterns, rendering them unsuitable for integrated circuit fabrication [139].

The template-assisted self-assembly approach is divided into top-down and bottom-up strategies. The top-down method pre-patterns or chemically modifies the substrate to create artificial trenches, guiding CNTs into parallel alignment due to spatial confinement. It enables wafer-scale arrays with a density of 50 SWCNTs/μm, but suffers from poor uniformity and orientation within trenches, low effective substrate coverage, and difficulties in processing below 10 nm [140]. The bottom-up method uses DNA brick crystals to construct parallel nano-trenches, with periodicity defined by programmable DNA repeat units. Single-stranded DNA handles are introduced at the trench bottoms to hybridize with anti-handles wrapped around CNTs via non-covalent interactions. This hybridization mediates CNT self-assembly within the nano-trenches, achieving densities exceeding 40 CNTs/μm with an orientation deviation < 2°. However, this method usually requires the use of photolithography or DNA templates for processing, and therefore faces the problems of high cost, low yield, and a complex and time-consuming process, and it is expected for it to be difficult to achieve commercial application in the short term [141].

The DLSA technique was proposed by the team from Peking University. DLSA involves immersing a substrate in a CNT solution and slowly withdrawing it; see Figure 12b for illustration [13]. During this process, CNTs are attracted to the two-dimensional solution–air interface, then transferred to the solid–liquid–gas contact line through the combined effects of substrate withdrawal and top-layer solvent evaporation convection, depositing continuously onto the wafer surface. By optimizing the two-phase interface and precisely controlling withdrawal speed, this method achieves CNT arrays with purity > 99.9999%, controllable density of 100–200 tubes/μm, orientation deviation < 8.3°, and diameters of 1.45 ± 0.23 nm. DLSA was the first to meet the s-CNT array requirements for high-performance electronics, enabling the fabrication of CNT transistors surpassing silicon-based counterparts [13]. Despite progress in scalable production, DLSA-prepared wafer-scale A-CNTs still exhibit alignment defects. In 2024, Bo Wang et al. conducted a systematic evaluation of CNT arrays prepared via the DLSA method using high-resolution transmission electron microscopy (HR-TEM). They found that only 9% of the pristine array exhibited ideal alignment, with the remainder consisting of bundles (46%), voids, misorientation, and stacking. The proportion of bundles after the micromanufacturing process increased to 60%, attributed to lateral CNT migration induced by metal/HfO_2_ shrinkage. Additionally, they also found that the average length of A-CNTs was shorter than the ideal value, and 89% of CNTs exhibited mutual contact (with contact coverage, *R*_CC_, ranging from 3% to 66%), potentially leading to additional parasitic capacitance. These findings highlight numerous unresolved issues in current array preparation. For very large-scale integrated circuit (VLSI) applications, CNT purity must also be improved by an additional 2–3 orders of magnitude [142]. Further purification of carbon nanotubes increases process steps and costs while reducing yield. Moreover, CNTs dispersed and aligned via solution-based methods are coated with polymers, which not only impair the contact performance of the source-drain electrodes in transistors but also affect gate dielectric deposition and interface states, potentially compromising the operational stability and uniformity of devices and circuits. To achieve consistent large-scale production, it is necessary to further improve the uniformity of orientation, spacing, and density. This requires precise control and processing of parameters such as solvent evaporation rate, substrate wettability, solution concentration and viscosity, solvent polarity matching, temperature gradient, surfactant residue, etc. Otherwise, the emergence of effects such as coffee rings will lead to problems such as the uneven orientation and density of carbon nanotubes. At the same time, the cleanliness and electrical quality of the material must be strictly characterized and controlled, and the cost must be reduced.

In a comprehensive comparison, both the CVD method and post-processing purification method face multiple intertwined challenges in achieving high purity and high density, including material selectivity, growth process control, post-processing efficacy, and large-scale scalability. Specifically, the CVD method typically satisfies only a subset of parameters, but fails to achieve wafer-scale production. Thus, there is a pressing need to develop methods that simultaneously meet all parameter requirements. In the post-processing purification method, Langmuir and evaporation-driven self-assembly struggle with poor density controllability and challenges in achieving uniform wafer-scale coverage, while also needing to prevent CNT stacking into bilayers or multilayers. Template-assisted self-assembly methods suffer from low yield, high cost, and the need to improve the stability of DNA-CNT interactions, requiring further development to meet the demands of industrialized carbon-based electronics. Currently, the DLSA method is the closest to meeting these requirements, but it still necessitates improved control over alignment, spacing, and density uniformity, as well as solutions to issues like CNT bundling.

### 5.2. Device Structure Optimization

#### 5.2.1. Scaling Down

In the realm of integrated circuit scaling, silicon-based CMOS devices have advanced into sub-5 nm process nodes. Continuous device miniaturization has introduced a series of physical and process-related challenges, including SCE, rising contact resistance, increased leakage current, power constraints, and escalating manufacturing costs. While technologies like FinFETs and GAA structures have mitigated the SCE to some extent, their fabrication complexity and cost have surged, driving the industry to explore new materials. Researchers have extensively studied CNT FETs’ performance benefits and scaling potential, achieving significant progress.

Early studies primarily focused on FETs with a single CNT to explore the ultimate electrical performance limits during gate length and contact length scaling. As early as 2004, Javey et al. from Stanford introduced high-k gate dielectrics (HfO_2_), self-aligned structures, and Pd metal source-drain contacts to fabricate p-type transistors with a channel length of 50 nm. These devices achieved a peak transconductance of 30 μS/tube, a saturation current of 25 μA (*V*_ds_ = 0.4 V), an SS of 110 mV/decade, and an on-state conductance of 0.5G_0_, approaching the theoretical limit for single-tube on-state current [143]. Subsequently, Franklin et al. from IBM systematically analyzed the significant impact of contact length (*L*_c_) on performance by constructing multiple transistors with varying channel and contact lengths on a single carbon nanotube [38]. They found that contact resistance increases markedly as *L*_c_ decreases, dominating in sub-10 nm devices. By employing an ultrathin gate dielectric (3 nm HfO_2_), they fabricated a device with a 9 nm channel length, achieving an *I*_on_ of 2410 μA/μm (*V*_ds_ = 0.5 V) bias, with an SS as low as 94 mV/decade, demonstrating the superior performance of sub-10 nm CNTFETs [25,38]. A milestone was reached in 2017 when Qiu et al. from Peking University successfully scaled the gate length of a CNTFET to 5 nm, achieving near-ballistic transport and single-electron switching. They also demonstrated a CMOS inverter with a total pitch of only 240 nm, more compact than silicon’s 22 nm node, providing critical evidence for the extreme scaling and practical circuit applications of CNTFETs [7].

While single CNTs are ideal for studying scaling limits and fundamental principles, they are impractical for large-scale ICs. Therefore, verifying the scaling capabilities of CNT arrays and developing corresponding processes are essential for realizing high-performance device applications. In 2017, Cao et al. utilized high-density A-CNTs to scale the device channel to 40 nm, achieving a current density of 0.8 mA/μm, surpassing that of silicon-based FinFETs at the 5 nm node [15]. However, these A-CNT-based devices exhibited gate control issues, with an SS of 500 mV/decade, significantly higher than that of single-tube devices, indicating substantial challenges at the oxide interface [15]. In 2023, the team from Peking University further advanced A-CNT FETs by scaling the gate length to 30 nm (contacted gate pitch of 54 nm) (see Figure 12c), equivalent to silicon’s 10 nm technology node, while maintaining high performance. They also constructed a static random-access memory (SRAM) cell using six A-CNT FETs, occupying an area of only 0.976 μm², comparable to the SRAM area of silicon’s 90 nm node, further demonstrating that A-CNT FETs can rival silicon-based technologies at ultra-small dimensions [33].

Currently, CNT FETs at ultra-small dimensions (e.g., 5 nm gate length) exhibit performance potential superior to silicon technologies. Theoretical studies further indicate that 5 nm represents the practical scaling limit for CNTFETs, with significant advantages in energy-delay product (EDP), *I*_on_, delay time (τ), and power dissipation (PDP) compared to two-dimensional materials and silicon FinFETs [22]. However, to achieve the practical application and industrialization of high-performance CNTFETs, future efforts must focus on synergistic advancements in improving contact performance, refining passivation processes, developing novel gate dielectrics, exploring GAA structure fabrication, and enhancing material preparation. These developments are critical to transitioning CNTFETs from the laboratory to real-world applications.

#### 5.2.2. Optimization of Gate Control Capability

The ultrathin body of carbon nanotubes endows them with excellent gate control efficiency; however, it also makes them more susceptible to surface charge interference. This heightened sensitivity to gate dielectric charge traps or random fixed charges has been quantitatively analyzed by researchers, who found that a single charge trap can induce significant random telegraph signal (RTS) noise in carbon nanotube field-effect transistors with amplitudes reaching 60% or higher, far exceeding the 5% observed in silicon-based MOSFETs [144]. Consequently, CNT devices require a cleaner gate dielectric to maintain performance stability.

**Challenges in Gate Dielectric Growth.** Conventional gate structures for CNTs include back gate, top gate, double gate, and gate-all-around. For CNT MOSFETs, fabricating a back gate does not require direct atomic layer deposition growth on the CNT surface, making it less process-intensive but offering lower electrostatic gate control efficiency [25]. Back-gate devices also typically exhibit larger *SS* and hysteresis, necessitating passivation processes to mitigate impurities like water and oxygen adsorbed on the CNT surface [145]. In contrast, top-gate structures theoretically provide superior gate capacitance and control, making them more suitable for high-performance devices. However, the absence of dangling bonds on the inert sp²-hybridized CNT surface, which lacks nucleation sites, poses challenges for ALD growth. Common methods to fabricate top gates include overgrowing the gate dielectric via ALD to encapsulate the CNTs or introducing a seed layer to assist ALD growth. High-k dielectrics such as HfO_2_ [34,37,146], ZrO_2_ [147], and La_2_O_3_ [148] are frequently used, while another approach involves forming Y_2_O_3_ through thermal oxidation of metal, which offers good wettability [149,150,151].

To address the lack of nucleation sites, researchers have explored techniques such as DNA modification, amorphous carbon seed layers, and chemical group bonding to introduce nucleation points [7,152,153]. However, these methods often suffer from poor uniformity and reliability. For instance, chemical modification typically introduces structural defects in CNTs, undermining their high-mobility advantage [154]. A more promising approach involves depositing an ultrathin intermediate dielectric layer to provide nucleation sites, followed by ALD growth of a uniform, high-quality high-k dielectric layer. Transistors with this dual-layer stacked structure have demonstrated exceptional gate control performance in existing studies. The intermediate layer must ideally exhibit a high dielectric constant k, wide bandgap, low defect density, and uniform growth on both the CNT and substrate surfaces to support ALD growth without compromising the material’s intrinsic properties. However, reported intermediate layer materials only partially meet these criteria: Al_2_O_3_ offers a large bandgap and ultrathin uniformity but has excessive defect states [14]; Y_2_O_3_ features relatively low interface defects but lacks uniform growth [151]; TiO_2_ faces similar limitations [155]. Physical vapor deposition (PVD) facilitates intermediate layer growth but struggles to maintain film thickness uniformity at nanoscale dimensions [151,155]. Some studies have attempted to achieve uniform intermediate layer deposition via CVD components, though these processes remain immature [14]. Overall, the intermediate dielectric layer process presents ongoing challenges, requiring systematic research to identify suitable materials and growth methods for CNT devices.

Double-gate and gate-all-around structures theoretically offer superior gate control but entail greater fabrication complexity, building on the foundation of robust top-gate processes. For example, D. Franklin implemented a self-aligned GAA structure on a single CNT, but due to suboptimal gate dielectric quality, its performance did not surpass that of existing top-gate devices [4].

**Selection of Gate Metal.** Ideal CMOS technology for CNT FETs is doping-free, precluding the traditional approach of adjusting *V*_th_ through channel doping, as used in silicon-based devices. Instead, *V*_th_ modulation relies on tuning the gate metal work function or gate dielectric fixed charges. Currently, precise control of fixed charges in the gate dielectric remains challenging and is thus unsuitable for *V*_th_ adjustment, making gate metal work function modulation a more practical choice. Reports have demonstrated that using Pd and Ti as gate metals can shift *V*_th_ by approximately 1 V [23]. For large-scale integrated circuits, continuous *V*_th_ tuning is often required. The team from Peking University achieved this by stacking Pd and Sc metals with different work functions, enabling continuous *V*_th_ adjustment from −1.0 V to 0.2 V [156]. Additionally, drawing from silicon-based high-k metal gate (HKMG) processes, alloy gates could be employed to achieve specific *V*_th_ values.

**Optimization of Gate Structure.** The small bandgap and low effective mass of CNTs, combined with the ambipolar nature of Schottky contacts in transistors, render CNTFET performance particularly susceptible to GIDL, resulting in higher static power consumption. Industry standards dictate off-state leakage currents not exceeding 100 nA/μm, 10 nA/μm, or 1 nA/μm for high-performance, standard-performance, and low-power applications, respectively. Thus, suppressing ambipolar behavior and reducing off-state leakage in CNT FETs is critical.

To address this, a team from Peking University developed a feedback gate (FBG) structure by adding a feedback gate near the drain, effectively suppressing leakage current and ambipolar behavior in CNT FETs. Their 500 nm top-gate devices achieved an *I*_off_ as low as 0.1 pA, an on–off ratio of eight orders of magnitude, and an *SS* of 75 mV/decade [157]. In 2019, the team validated FBG effectiveness at sub-400 nm channel lengths in CNT network films. As channel length decreases, the proportion of the gap (63 nm) between the feedback gate and main gate increases, potentially weakening gate control [158]. In 2020, the team introduced a strengthened CMOS (SCMOS) architecture, incorporating an assistant gate in FET designs to optimize both on- and off-state performance. The SCMOS inverter reduced static power consumption to 100 nW, three orders of magnitude lower than traditional CMOS technology, while the SCMOS ring oscillator achieved a maximum frequency of 152.8 MHz, a 50% improvement over the 104.4 MHz of conventional CMOS ring oscillators. Scalability was further demonstrated through 7-stage and 12-stage inverter chains, NAND, and NOR gates [159].

However, FBG and split-gate structures struggle to maintain effectiveness under aggressive scaling, limiting their applicability to advanced nodes. To overcome this, in 2021, a team from Peking University, building on FBG principles, experimental data, and TCAD simulations, proposed an L-shaped sidewall CNTFET design conducive to scaling and large-scale integration; see Figure 12d for the schematic. By applying the drain potential directly to an L-shaped spacer near the drain, this design widens the off-state barrier, suppressing GIDL-induced leakage. Unlike FBG, the L-shaped spacer employs a simple self-aligned process, avoiding lift-off process limitations and enhancing manufacturability. Simulations showed that at a 50 nm gate length, *I*_off_ dropped to ~1 nA/μm, which is two orders of magnitude lower than conventional structures [131]. Overall, devices based on the FBG principle require further optimization and experimental validation in short-channel performance, process complexity, material selection, and power efficiency to fully realize their potential.

#### 5.2.3. Contact Optimization

The performance of CNT FETs is constrained by the contact resistance between the source/drain electrodes and the CNTs. Unlike silicon-based CMOS devices, the quasi-one-dimensional nature of CNT FETs makes them highly sensitive to the contact interface. Ideally, contacts should meet the following criteria: (1) Form low-resistance ohmic contacts without Schottky barriers, with appropriate electrode materials tailored for n-type and p-type devices; (2) Ensure chemical stability at the metal–CNT interface to prevent carrier scattering due to defects. The team from Peking University has developed PMOS devices with Pd contacts and NMOS devices with Sc or Y contacts, achieving symmetric electrical characteristics for p-type and n-type devices. Both hole and electron mobilities exceed 3000 cm^2^·V^−1^·s^−1^, with on-state conductance reaching 0.6G_0_, approaching the quantum conductance limit of metal–semiconductor CNT contacts [12,34,35,36]. However, unlike devices based on a single CNT, A-CNT devices face greater challenges in contact processing. In particular, it has long been difficult for the performance of A-CNT n-type FETs to match that of A-CNT p-type FETs. To address this challenge, researchers employed hydrophobic substrate treatment techniques to successfully fabricate high-performance A-CNT N-FETs. This breakthrough not only bridged the gap for A-CNT in CMOS technology but also enabled an in-depth study of their scaling characteristics [39,160]. In developing n-type devices, the use of low-work-function contact metals also presents significant hurdles. These metals oxidize readily during low-vacuum deposition or upon air exposure, resulting in device failure or a sharp increase in contact resistance. This issue severely limits the performance and reliability of NMOS devices. Consequently, encapsulating Sc contacts to isolate them from moisture and oxygen is crucial. Reports have shown that covering the top and sidewalls of contact electrodes with an Al_2_O_3_ passivation layer effectively shields them from environmental effects, maintaining device stability after 146 days of air exposure [161]. We believe that advanced industrial encapsulation techniques could further enhance NMOS contact stability. Beyond encapsulation, researchers have explored chemical or electrostatic doping to improve n-type device performance, but these methods suffer from poor stability and degraded transistor performance, making them unsuitable for high-performance applications [162,163,164].

Unlike planar contacts in bulk materials, CNT transistor contacts typically involve metal wrapping around three sides of the nanotube. Due to capillary forces and vdW interactions during metal deposition, CNTs are susceptible to deformation or collapse. Axial symmetry breaking and wavefunction mismatch between collapsed and circular segments increase contact resistance by threefold. Even in fully collapsed CNTs, band splitting from π-orbital interactions further elevates resistance [165]. Additionally, CNT displacement or clustering during evaporation can impact device uniformity and performance. Studies also reveal that metal wrapping morphology varies with CNT spacing. As array pitch decreases, metal contacts undergo a first-order wetting transition: at larger pitches, metal wets the substrate between CNTs; at smaller pitches, it suspends atop the CNT array, resembling a ‘lotus leaf effect’. The critical pitch for this transition ranges from 4 to 12 nm, depending on substrate–metal interaction strength and CNT diameter. In the non-wetting state, a reduced metal–CNT contact area weakens electrical coupling, increasing contact resistance and transfer length, posing challenges for device miniaturization and high-density integration [166].

**Figure 13:**
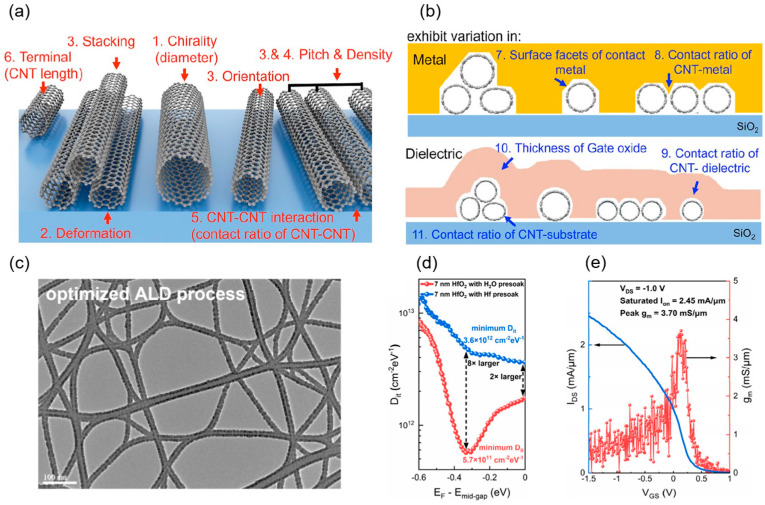
Non-ideality in (**a**) A-CNT arrays and (**b**) A-CNT FETs. Reprinted with permission from [142]. Copyright 2024 American Chemical Society. (**c**) TEM image of optimized HfO_2_ dielectric grown on A-CNTs through H_2_O soak. Reprinted with permission from [167]. Copyright 2024 American Chemical Society. (**d**) Interface state density with H_2_O or Hf presoak [43]. Reprinted with permission from IEEE Proceedings. (**e**) Transfer characteristics and transconductance curve of optimized A-CNT FET with *L*_G_ of 100 nm [43]. Reprinted with permission from IEEE Proceedings.

Systematic characterization using HR-TEM of A-CNT FETs has confirmed these issues. Included in Figure 13a,b are 11 nonideal scenarios existing in A-CNT arrays and FETs. In high-density CNT arrays prepared via DLSA, most CNTs exhibit significant radial strain (some exceeding 20%) in metal-contact (Pd-covered) and channel (HfO_2_-covered) regions, with complex and variable strain fields. Moreover, CNT–metal contact ratios are highly inconsistent, averaging 40.2% for Y contacts with a maximum of 84.6% [142]. Such phenomena are rare in bulk material devices, highlighting the unique challenges in CNTFET design. Contact optimization must balance deformation and electrical performance, necessitating refined metal and dielectric deposition processes to improve contact ratio and uniformity.

#### 5.2.4. Interface States

Due to their quasi-one-dimensional nature, CNTFETs are highly sensitive to interface quality. Unlike silicon-based MOSFETs, the channel in CNT FETs consists of sp²-hybridized carbon atoms with no dangling bonds on the surface, theoretically suggesting a low interface state density. However, in practical devices, interactions with the gate dielectric and contact electrodes introduce numerous interface states. These states lead to non-ideal gate control efficiency, which impairs gate modulation and *V*_th_ tuning. Additionally, interface defects and impurities decrease carrier mobility, with experimental values typically less than 5000 cm^2^·V^−1^·s^−1^ compared to a theoretical value exceeding 20,000 cm^2^·V^−1^·s^−1^, causing the on-state current to drop well below the theoretical ballistic limit. They also weaken electric field control, exacerbating the SCE, manifesting as increased *SS*, reduced transconductance, and decreased on–off ratio. This severely impacts device energy efficiency and long-term stability. Unlike silicon-based MOS devices, the origins of interface states in A-CNT MOS structures are complex and lack systematic study. Thus, understanding the sources and mechanisms of interface states and developing effective optimization strategies are critical for the industrialization of A-CNT MOSFETs.

Early studies focused on the effects of water and oxygen adsorption at the silicon oxide back-gate interface on device performance. Researchers reduced hysteresis in CNT FETs by minimizing water and oxygen adsorption on the CNTs and SiO_2_ surfaces [153,168]. Alternatively, wet etching was used to refresh the Si/SiO_2_ substrate surface, removing residual polymers and releasing stress at the interface between A-CNTs and the substrate, thereby enhancing the performance of A-CNT field-effect transistors [169]. In 2016, Rebecca Sejung Park et al. employed pulsed time-domain measurement (PTDM) techniques to quantitatively analyze hysteresis in CNT FETs, extracting trap density, energy distribution, and spatial distribution for the first time. They calculated the interface trap density at the CNT-SiO_2_ interface in top-gate devices to be approximately 3 × 10^13^ cm^−2^·eV^−1^, which is one to two orders of magnitude higher than that of traditional silicon-based CMOS interfaces. They further reduced hysteresis by shrinking the equivalent oxide thickness to enhance the electrostatic screening of the gate electrode, mitigating the impact of interface and surface traps on CNTs. However, this study did not deeply explore the specific sources of interface fixed charges or their mechanisms affecting *V*_th_ [170,171].

For the lack of quantitative analysis on the physical origins of interface states in A-CNTs, in 2024, Yifan Liu et al. from Peking University utilized admittance spectroscopy to precisely measure C-V and G-V characteristics, combined with density functional theory (DFT) calculations [167]. This work marked the first systematic study of interface states and border trap density in A-CNT MOS structures, revealing their physical origins. They identified microscopic defects at the CNT-HfO_2_ interface, including dangling bonds, oxygen vacancies (V_O_), and interface contaminants. These defects form during the ALD growth of HfO_2_, particularly in low-temperature processes (<250 °C). DFT analysis showed that oxygen vacancies in HfO_2_ on the A-CNT surface are primary sources of interface states, introducing additional energy levels within the bandgap that cause charge trapping. Analysis of the energy distribution of interface states via C-V and G-V revealed a broad distribution across the A-CNT bandgap, with particularly high density near the midgap (on the order of 10^12^–10^13^ cm^−2^·eV^−1^), which is two to three orders of magnitude higher than that in silicon-based MOS devices (where *D*_it_ is typically ~10^10^ cm^−2^·eV^−1^). To tackle this, they employed an oxygen-enhanced ALD process to grow HfO_2_, improving its morphology (see Figure 13c) and reducing oxygen vacancies. This reduced *D*_it_ to 6.1 × 10^11^ cm^−2^·eV^−1^, representing the lowest recorded *D*_it_ for low-dimensional semiconductor MOSFETs. Combined with an air-gap spacer design, they lowered the *I*_off_ from 300 nA/μm to 10 nA/μm and drain-induced barrier lowering (DIBL) from 300 mV/V to 75 mV/V, while maintaining a high *I*_on_ of 1.48 mA/μm at *L*_g_ of 80 nm and *V*_ds_ of 1 V. Based on continuous ALD process optimization, they again reduced *D*_it_ to 5.7 × 10^11^ cm^−2^·eV^−1^, see Figure 13d, featuring saturated on-current of 2.45 mA/μm and record transconductance of 3.7 mS/μm (see Figure 13e), far surpassing the maximum *g*_m_ value of Si planar FETs. Although *D*_it_ was reduced to 5.7 × 10^11^ cm^−2^·eV^−1^, further optimization of *D*_it_ to below 1 × 10^11^ cm^−2^·eV^−1^ is needed to achieve greater device energy efficiency and uniformity [43,167]. Future improvements could involve refining dielectric morphology, minimizing microscopic defects, and converting reactive defects into inert structures to further lower *D*_it_.

### 5.3. Challenges to the Integration

Currently, the fabrication of CNT devices remains largely confined to laboratory research, relying heavily on electron beam lithography (EBL) and lift-off techniques for pattern transfer. These methods enable electrode definition with sub-100 nm precision. Additionally, self-aligned processes (e.g., ALD-grown HfO_2_ as the gate dielectric) can reduce alignment errors between the gate and source/drain to below 5 nm, enhancing device performance uniformity. For instance, the team from Peking University utilized EBL to fabricate CNT FETs with a 5 nm gate length [7].

However, many laboratory processes are unsuitable for scalable production. The lift-off process currently required for patterning lacks scalability at submicron nodes. For instance, the photoresist (PR) used in this process has a temperature tolerance limit of approximately 200 °C, whereas the ALD and post-deposition annealing of high-quality gate dielectrics (e.g., HfO_2_) demand temperatures above 250 °C. Higher temperatures help reduce defects, fixed charges, and interface states, but the low-temperature constraint of PR restricts dielectric growth conditions. During the lift-off process, the thin film undergoes random deformation, such as edge curling and burrs, when the PR is removed, leading to morphological defects that degrade device performance consistency and reliability. Additionally, CNTs exposed to PR and its solvents during lift-off may suffer from chemical contamination and physical displacement. For n-type CNT FETs using low-work-function metals as contact materials, oxygen or water vapor can infiltrate the Sc-CNT interface during the lift-off process, forming an oxide layer that shortens the effective contact length and causes device failure. These shortcomings of the lift-off process stem from its reliance on the low-temperature properties of PR and the imprecision of mechanical removal, limiting its applicability in high-temperature dielectric growth, structural optimization, and precise patterning. Consequently, dry etching processes are essential for large-scale integration of small-sized, high-performance CMOSs [172]. However, CNTs are sensitive to certain plasma elements, and conventional dry etching can introduce lattice defects and performance degradation. This necessitates the optimization of etching gases (e.g., CF_4_/O_2_ mixtures) and process parameters, rational design of etch-stop layers, precise control of etch rates, or adoption of advanced industrial atomic layer etching techniques. Moreover, due to the sensitivity of CNT arrays as low-dimensional materials, every processing step must prevent contamination or damage. To protect CNTs, developing a sacrificial layer that can shield the CNT layer during fabrication and be removed without leaving organic or metallic residues is essential. Clearly, the practical application and industrialization of carbon-based electronics face numerous process compatibility challenges. Addressing these challenges requires integrating the physicochemical properties of CNTs into the design of compatible fabrication processes, which demands collaboration between research institutions and industry. Encouragingly, this goal is not unattainable. The MIT Shulaker group has made pioneering efforts toward industrialization by optimizing CNT deposition methods to meet commercial manufacturing requirements. Their work successfully demonstrated the fabrication of 14,400 CNT FETs with 100% yield on a 200 mm silicon wafer using commercial silicon foundries (Analog Devices, Inc., Wilmington, MA, USA and SkyWater Technology, Bloomington, MN, USA) [173].

## 6. Future Development Directions for High-Performance CNT Devices

### 6.1. Material Optimization and Fabrication Techniques

As one of the most promising semiconductor materials in the post-Moore era, the optimization of carbon nanotube materials and their fabrication techniques forms the critical foundation for achieving high-performance devices. To meet the demands of advanced integrated circuit nodes, ideal CNT arrays must exhibit ultra-high semiconducting purity, uniform tube diameter, controllable density, uniform pitch and length, and directional alignment. Specific benchmarks can be referenced, as outlined by Yumeng Ze [127]. For ultra-large-scale integrated circuits with stringent performance and uniformity requirements, considering silicon-based technology’s tolerance for metal impurities (silicon wafer metal impurities < 1 × 10^10^ atoms/cm^2^), the semiconducting purity of CNTs needs to improve by 2–3 orders of magnitude. Simultaneously, array alignment methods must be refined, and low-cost, highly controllable solution-based self-assembly techniques (e.g., DLSA) should be developed to achieve full coverage and directional alignment on 8-inch or even 12-inch wafers. This process should also minimize lattice damage and chemical residues during fabrication to enhance carrier mobility (target > 1500 cm^2^·V^−1^·s^−1^); see Table 2. Specifically, we propose potential optimization techniques based on the two strategies for the preparation of CNT arrays. The preparation processes involve precise control and coordination of material interactions at the nanoscale; therefore, the complex dynamics of material growth and interactions must be comprehensively considered during optimization. For the CVD method, we suggest that further improvements can be achieved in the following three aspects, building upon the promising results obtained in the growth of single-chirality CNTs on hBN substrates [44]. In terms of substrate selection, other two-dimensional materials (such as graphene, transition metal sulfides, etc.) can be explored as substrates in the future to further optimize interface interactions and explore the process of preparing wafer-level atomic-level flat interfaces to meet the requirements of large-scale integrated circuits. In catalyst design, developing highly active, long-lifetime solid-state alloy catalysts with optimized size distributions can enhance chirality selectivity and growth rates. For growth conditions, optimizing flow and temperature field distributions, adjusting gas ratios, and dynamically tuning growth parameters can achieve more uniform array density. At the same time, first-principles and molecular dynamics theory simulations and mechanism studies can be combined, and machine learning as well as other methods can be introduced to more accurately predict array growth dynamics. For the template-assisted self-assembly method, we believe that low-cost patterning technologies such as nanoimprint need to be developed in the future to achieve wafer-level preparation. At present, the DLSA method is the most promising method to achieve the ideal CNT array in the short term. In the future, machine learning can be used to optimize DLSA parameters such as pulling rate and liquid film thickness, temperature gradient design, solvent ratio, and interface tension control on the basis of improving the purity of the solution. Furthermore, the DLSA method can be combined with the template-assisted self-assembly method to use a pre-patterned substrate to achieve density-spacing decoupling to further improve the purity, density, and uniformity of the CNT array. These breakthroughs require interdisciplinary collaboration that integrates materials, fluid mechanics, and semiconductor processes to promote their transition from the laboratory to industrialization.

### 6.2. Device Structure and Process Innovations

At the device level, CNTs require breakthroughs in several key areas: optimizing end contact (e.g., metal carbides) to reduce contact resistance (target < 232 Ω at 18 nm contact length, see Table 2) while addressing the stability issues of n-type contacts. Exploration of new low-work-function metals or alloys is needed to mitigate NMOS electrode oxidation. For gate design, developing ultrathin high-k gate dielectrics (e.g., HfO_2_/Y_2_O_3_ stacks) is essential to lower interface state density (target < 10^11^ cm^−2^·eV^−1^). Research into GAA structures should be advanced to improve gate control efficiency and suppress short-channel effects. Additionally, L-shaped sidewall designs should be studied to reduce off-state leakage current (target < 1 nA/μm) and enhance the on–off ratio (>10^6^).

In terms of process compatibility, to reduce production costs, CNT device fabrication must quickly align with silicon-based processes. This involves developing dry etching, ALD, and other silicon-compatible techniques while addressing CNT transfer and protection challenges. The process must prevent contamination of CNTs by silicon-based fabrication steps.

### 6.3. Performance Limit Breakthroughs

For high-frequency and low-power CNT devices, efforts should focus on pushing radiofrequency transistors into the terahertz range by optimizing T-shaped or U-shaped gate structures to reduce parasitic effects. In novel transistor development, further exploration of DSFETs and other architectures is needed to achieve *SS* below 60 mV/decade and operating voltage under 0.5 V.

In three-dimensional integration, monolithic 3D integration solutions should be investigated, combining high-density vias with low-temperature processes to enhance system energy efficiency.

### 6.4. Expansion of Application Scenarios

Digital Integrated Circuits: achieve ultra-large-scale carbon nanotube-based CMOS integration (>10^5^ transistors) and validate the commercial potential of high-performance processors (e.g., RISC-V architecture).

Radiofrequency Electronics: develop K/Ka-band power amplifiers with improved linearity (OIP3/P_dc_ > 20 dB) and output power (>10 dBm).

Integrated Sensing Platforms: integrate biological/gas sensors with signal processing circuits to create multifunctional IoT nodes capable of single-molecule-level detection sensitivity.

### 6.5. Addressing Industrialization Challenges

Develop device-circuit models for CNT-based systems and a comprehensive process design kit (PDK) to serve as a bridge between IC design companies, foundries, and EDA vendors. Establish standards for A-CNT thin-film materials, including substrate type, semiconducting purity, array density, tube diameter and length distribution, alignment distribution, defect density, sheet resistance distribution, metal ion content, surface polymer content, and other metrics reflecting material integrity. Provide measurement methods, reference ranges, and recommended instruments for these parameters.

### 6.6. Roadmap for Industrialization

(1) Short-Term Goals
  Specialized Chips: Enable practical use in radiation-resistant circuits (aerospace), low-temperature circuits, flexible electronics, display drivers and biosensors, etc. At present, CNT-based sensors such as hydrogen sensors have been commercialized.
(2) Mid-Term Goals
  High-Frequency RF Modules: partially replace GaAs and InP devices, targeting applications in 6G base stations.
(3) Long-Term Goals
  Logic Circuit Replacement: replace silicon-based CMOS technology at 3 nm and below nodes, capturing the high-end chip market.

## 7. Summary and Outlook

CNT electronic devices, with their exceptional properties such as high carrier mobility and intrinsic low power consumption, have emerged as a pivotal research direction for semiconductor technology in the post-Moore era, offering a new breakthrough for industrial development. Extensive fundamental research has validated their immense potential across diverse applications. This review systematically summarizes the intrinsic material advantages of CNTs and provides a comprehensive overview of their advancements in field-effect transistors, radio-frequency devices, logic circuits, brain-inspired neuromorphic devices, and optoelectronic integration. Experimental results demonstrate that CNT devices exhibit the potential to surpass traditional silicon-based CMOS technology in critical metrics such as speed, power consumption, and integration density. Notably, their advantages in novel device paradigms, such as ternary logic and Dirac-source transistors, offer viable alternative solutions and expansive prospects for semiconductor technology. Looking forward, CNT-based high-performance ICs are poised to drive technological breakthroughs in multiple domains, including ultra-high-speed digital circuits, carbon-based RF front-ends, 3D monolithic integration, on-chip optoelectronic systems, neuromorphic computing architectures, ultrasensitive sensors, flexible wearable electronics, and multifunctional specialized chips. These advancements will not only enable large-scale data processing and complex computations but also find broad applications in IoT, wearable devices, 6G communications, autonomous driving, and intelligent systems. By deeply integrating with emerging technologies such as deep learning, quantum computing, and artificial intelligence, CNT-based ICs are expected to accelerate innovation in intelligent computing and next-generation applications, laying a robust foundation for the evolution of post-Moore electronics.

However, the practical deployment of high-performance CNT electronic devices still faces significant challenges. To fully harness the potential of CNTs in ICs, sustained research efforts are required in material synthesis, device fabrication, system integration, and industrialization. At the material level, scalable production of high-purity semiconducting CNTs remains a hurdle. Developing chirality-selective growth techniques, high-throughput nondestructive separation and purification methods, and assembly processes that meet the stringent requirements for CNT array parameters (e.g., purity, density, alignment) is essential. In terms of device fabrication and process optimization, there is an urgent need to address challenges such as contact resistance, interface state engineering, and high-quality gate dielectric growth, alongside exploring innovative device designs, including the optimization of novel architectures like Dirac-source transistors. For system integration, advancements in scaling-down processes and circuit design must be complemented by exploring heterogeneous integration approaches, such as Chiplet technology, to enable multifunctional module integration. For 3D integration, innovative architectures like CMOSs, complementary FETs, and Flip FETs, coupled with advanced interconnect technologies and thermal dissipation management, are critical to achieving high-density, high-performance 3D ICs. On the industrialization front, improvements in yield, cost reduction, establishment of standardized process flows compatible with silicon-based technologies, and development of testing platforms and electronic design automation (EDA) tools are imperative. In the realm of analog circuits, future research should focus on optimizing T-shaped or Y-shaped gate structures in CNT-based RF devices, reducing channel resistance, and advancing system-level integration with components such as antennas, amplifiers, and modulators to construct RF-integrated circuits (RFICs). Furthermore, continued exploration of CNTs in neuromorphic computing, optoelectronic integration, and ternary logic is necessary to broaden their application scope. Moving forward, the advancement of CNT technology will require collaborative efforts across academia, industry, and research institutions, driving innovation across the entire ‘materials–devices–integration’ chain to gradually build a complete industrial ecosystem for CNT-based electronics.

## Figures and Tables

**Table 1:** The development of CNT FETs based on key metrics.

Ref.	Gate Type	*L*_ch_ (nm)	*V*_gs_ (V)	*V*_ds_ (V)	*I*_on_	*SS* (mV/dec)	*g*_m_	Notes	Year
[11]	BG SiO_2_ 67 nm	300	−7.5	−2.4	25 μA/tube	170	/	Single tube Pd contact	2003
	BG SiO_2_ 67 nm	3500	−5.5	−0.7	25 μA/tube	150	/		
[12]	BG SiO_2_ 100 nm	300	−2	0.5	20 μA/tube	250	/	Single tube Sc contact	2007
[37]	TG HfO_2_ 15 nm	120	1.0	1.0	25 μA/tube	100	25 μS	Single tube Sc contact	2008
[38]	LBG HfO_2_ 10 nm	20	*V*_LBG_ − *V*_th_ = −1	−0.5	10 μA/tube	85	20 μS	Single tube	2010
[4]	GAA AlO_x_N_y_ 1.5 nm + Al_2_O_3_ 5 nm	20	*V*_gs_ − *V*_th_ = −0.4	−0.4	~2 μA/tube	85–140	/	Single tube Pd contact	2013
	GAA AlO_x_N_y_ 1 nm + HfO_2_ 8 nm	30	*V*_gs_ − *V*_th_ = 0.4	0.4	~2 μA/tube	95–150	/	Single tube Pd contact	
[15]	TG Al_2_O_3_ 5 nm	11	−0.5	−0.5	3μA/tube	85	/	Single tube Co-Mo end contact	2017
	BG HfO_2_ 3 nm	~40	−3	−0.5	800 μA/μm	~500	0.32 mS/μm	CNT array Co-Mo end contact	
[7]	TG HfO_2_ 3.5 nm	5	−1	−0.1	2 μA/tube	73	55 μS	Single tube Graphene contact	2017
	TG HfO_2_ 3.5 nm	10	−1	−0.1	6 μA/tube	60	/	Single tube Graphene contact	
	TG HfO_2_ 3.5 nm	20	−1.25	−0.4	17.5 μA/tube	70	/	Single tube Pd contact	
	TG HfO_2_ 3.5 nm	20	0.6	0.4	~10 μA/tube	70	/	Single tube Sc contact	
[13]	TG HfO_2_ 7.3 nm	120	−2.5	−1	1300 μA/μm	100–200	0.9 mS/μm	CNT array Pd contact	2020
	Ionic-liquid	290	−1.2	−0.1	50 μA/μm	75–90			
[23]	TG HfO_2_ 4.8 nm	200 nm	−2	−1	1180 μA/μm	73	1.0 mS/μm	CNT array Pd contact	2021
[39]	TG HfO_2_ 10 nm	300	4	2	600 μA/μm	100	250 μS/μm	CNT array Sc contact	2022
	TG HfO_2_ 10 nm	100	4	3	800 μA/μm	170	250 μS/μm		
[33]	TG HfO_2_ 5 nm	30	−2.6	−0.7	3310 μA/μm	/	2.69 mS/μm	CNT array Pd contact	2023
	TG HfO_2_ 5 nm	85	−2	−0.8	2240 μA/μm	175	1.64 mS/μm		
[40]	GAA (Nanosheet) Al_2_O_3_ 1 nm + HfO_2_ 5 nm	70	*V*_gs_ − *V*_th_ = −0.7	−0.5	1150 μA/μm	135	~1.5 mS/μm	CNT array Pd contact	2024
		30	*V*_gs_ − *V*_th_ = −0.7	−0.5	~2000 μA/μm	~170	~2.5 mS/μm		
[41]	TG Al_2_O_3_ 4 nm	250	~−2.5	−1	220 μA/μm	200	/	CNT array Pd contact WO_x_ doping	2024
		250	~2.5	1	220 μA/μm	200	/	CNT array Ti contact AlN_x_ doping	
[42]	TG HfO_2_ 5 nm	200	−1	−1	1170 μA/μm	115	1.83	CNT array Pd contact Glass substract	2024
		300	−1	−1	830 μA/μm	95	1.34 mS/μm		
[43]	TG HfO_2_ 5 nm	100	−1.5	−1	2450 μA/μm	100	3.7 mS/μm	CNT array Pd contact	2024
[44]	BG SiO_2_ 285 nm	400	/	8	6500 μA/μm	/	/	CNT array Pd contact	2025

**Table 2:** Future development directions and key challenges for high-performance CNT devices.

Area	Key Goals/Metrics	Key Technologies and Challenges
Material Optimization	Semiconductor purity >99.9999%. Uniform control over tube diameter, length, density, and pitch. Wafer-scale aligned arrays (8~12 inch).	Develop low-cost self-assembly techniques with high controllability. Minimize impurity residues and lattice damage.
Device Architecture and Process Innovation	Contact resistance < 232 Ω (at 18 nm contact length). Interface trap < 10^11^ cm^−2^·eV^−1^. Off-state leakage current < 1 nA/μm and on–off ratio >10^6^.	Optimize contacts and interfacial quality. Implement GAA, L-shaped spacer, and new architectures.
Performance Limit Breakthroughs	RF transistor *f*_T_/*f*_MAX_ exceeding terahertz. *SS* < 60 mV/dec and operating voltage ≤ 0.5 V.	Optimize gate structures to reduce parasitics. Explore new device structures.
Integration and 3D Architectures	Larger-scale monolithic 3D integration with higher performance. High-density vertical interconnects and low-temperature processes.	Develop reliable low-temperature interconnect technologies. Advanced thermal management and interlayer interference mitigation.
Expansion of Application Scenarios	Digital circuits with integration of over 10^5^ devices. RF amplifiers with OIP3/P_dc_ > 20 dB and output power > 10 dBm. Sensor platforms with single-molecule detection sensitivity.	Validation of high-performance processors. Design of RF circuits. Integration of bio/gas sensing front-ends.
Standardization and Industrialization	Establishment of PDK design kits and standard device libraries. Definition of A-CNT thin film material standards. Standardized process techniques.	Co-development of model libraries with EDA vendors and foundries. Specifying material parameters, measurement and instrumentation.
